# Enhanced nucleosome assembly at CpG sites containing an extended 5-methylcytosine analogue

**DOI:** 10.1093/nar/gkac444

**Published:** 2022-06-01

**Authors:** Miglė Tomkuvienė, Markus Meier, Diana Ikasalaitė, Julia Wildenauer, Visvaldas Kairys, Saulius Klimašauskas, Laura Manelytė

**Affiliations:** Institute of Biotechnology, Life Sciences Center, Vilnius University, Vilnius LT-10257, Lithuania; Biochemistry III, University of Regensburg, Regensburg, Bavaria, DE-93053, Germany; Institute of Biotechnology, Life Sciences Center, Vilnius University, Vilnius LT-10257, Lithuania; Biochemistry III, University of Regensburg, Regensburg, Bavaria, DE-93053, Germany; Institute of Biotechnology, Life Sciences Center, Vilnius University, Vilnius LT-10257, Lithuania; Institute of Biotechnology, Life Sciences Center, Vilnius University, Vilnius LT-10257, Lithuania; Biochemistry III, University of Regensburg, Regensburg, Bavaria, DE-93053, Germany

## Abstract

Methylation of cytosine to 5-methylcytosine (mC) at CpG sites is a prevalent reversible epigenetic mark in vertebrates established by DNA methyltransferases (MTases); the attached methyl groups can alter local structure of DNA and chromatin as well as binding of dedicated proteins. Nucleosome assembly on methylated DNA has been studied extensively, however little is known how the chromatin structure is affected by larger chemical variations in the major groove of DNA. Here, we studied the nucleosome formation *in vitro* on DNA containing an extended 5mC analog, 5-(6-azidohex-2-ynyl)cytosine (ahyC) installed at biological relevant CpG sites. We found that multiple ahyC residues on 80-Widom and Hsp70 promoter DNA fragments proved compatible with nucleosome assembly. Moreover, unlike mC, ahyC increases the affinity of histones to the DNA, partially altering nucleosome positioning, stability, and the action of chromatin remodelers. Based on molecular dynamics calculations, we suggest that these new features are due to increased DNA flexibility at ahyC-modified sites. Our findings provide new insights into the biophysical behavior of modified DNA and open new ways for directed design of synthetic nucleosomes.

## Graphical Abstract

**Graphical Abstract:**
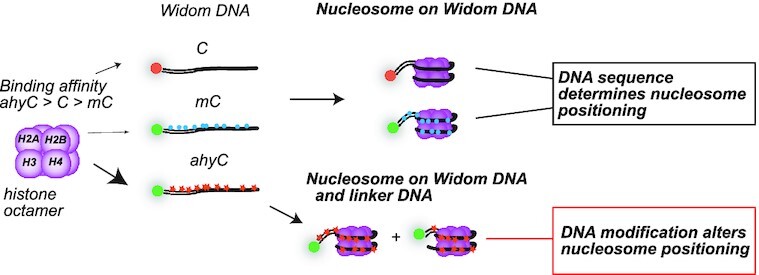
Extended 5mC analog, 5-(6-azidohex-2-ynyl)cytosine (ahyC) installed at biological relevant CpG sites is compatible with nucleosome assembly and attracts nucleosome to multiply modified sites.

## INTRODUCTION

Chromatin is a dynamic and multi-layered structure whose primary function is the packaging of the fibrous chromosomal DNA in the nucleus of an eukaryotic cell. The fundamental organizing units of chromatin are the nucleosomes, which consist of 147 bp DNA wrapped around the histone octamer comprising two copies each of the four highly conserved histone proteins H2A, H2B, H3 and H4 (1) and are separated from each other by 20–80 bp stretches of linker DNA. Distribution of the linker and nucleosome regions with regards to specific genetic loci may dramatically alter their accessibility to transcription, replication, DNA repair or recombination (2–5). On the other hand, active restructuring of the chromatin upon activation of DNA-dependent processes can be achieved by altering the nucleosome architecture and positioning by dedicated chromatin remodeling enzymes (6). These proteins belong to a superfamily II of ATPases and are responsible for repositioning, ejection or incorporation of nucleosomes on the DNA. Snf2H, one of the 53 human remodeling enzymes, functions as a catalytic subunit in several human chromatin remodeling complexes, such as ACF, CHRAC, WICH, RSF, NoRC and BRF5 (7,8). Thus, in addition to the DNA packaging role, the nucleosomes are essential regulators of all DNA-dependent processes.

Due to the helical nature of the DNA and the winding of DNA around the inner protein core, the nucleosome - DNA contacts of the histone octamer appear at 10 bp intervals and occur between the positively charged amino acids and the phosphates of the DNA backbone (9). Although generally, nucleosomes can form on almost any DNA sequence, some are more preferred than others. DNA sequences with AA, TT or TA dinucleotides spaced 10 bp apart possess intrinsic DNA curvature and show a higher affinity for the histone octamer. One such example is the Widom 601 DNA sequence, selected from *in vitro* Selex experiments (10).

Apart from the nucleotide sequence and active remodelers, nucleosome positioning is modulated by epigenetic modifications. Methylation at the 5th position of the cytosine ring at CpG sites is an epigenetic modification most often associated with gene silencing. *In vitro* experiments have shown that although 5mC makes DNA more rigid, methylated DNA can still be wrapped into nucleosomes (11). In mammals, DNA methylation occurs at 70–80% of CpG dinucleotides which are underrepresented throughout the genome (12). On the other hand, DNA regions with a high content of CpG dinucleotides, called CpG islands, are both hypomethylated and nucleosome-depleted in normal somatic cells (13) but may become hypermethylated and nucleosome-packed in the promoters of tumor suppressors in cancer cells (14). Interestingly, in contrast to mC, 5-hydroxylmethyl-cytosine (hmC), a product of TET enzyme activity on mC, is associated with labile nucleosomes (15). Understanding the dynamic structure of a nucleosome is key to the elucidation of genome packaging in eukaryotes, which is tied to the mechanism of gene regulation.

Synthetic nucleosomes may lead towards novel synthetic epigenetic marks. So far limited success in enhancing nucleosome formation has been achieved via certain histone modifications (amino acid substitutions, histone tail deletion or post-translational acetylation, methylation) (16). Such nucleosomes can be instrumental to study the function of the modifications and their impact on particular protein binding and action *in vitro*. Installation of the naturally occurring methyl- or other groups at the fifth position of cytosine in DNA appears to show a slight negative (mC) or positive (hmC) effect, which modulates binding but seems to lack the power required for repositioning of nucleosomes on DNA. To date, scarce data is available on using any unnatural DNA modifications to enhance nucleosome formation and stability either. Therefore, targeted nucleosome installation at predefined genomic sites remains outside reach without changes of the genomic sequence itself.

In this work, we sought to examine the effect of larger chemical variations in the major groove of the DNA helix on nucleosome formation. For this, we extended the biological methyl of mC at CpG sites to produce a linear moderately flexible, and moderately polar uncharged group. We selected the 6-azidohex-2-ynyl modification (ahyC), which can be installed in a sequence-specific manner using an engineered DNA methyltransferase reaction (17) and has proven particularly robust, versatile, and useful in numerous applications (18–23). We prepared a series of DNA substrates containing methyl- or azidohexynyl- groups at CpG sites and compared their capacity to assemble nucleosomes in direct competition with unmodified DNA. The resulting nucleosomes were further tested in thermal and ethidium bromide stability as well as nucleosome repositioning with the chromatin remodelers assays. Based on these experiments and molecular dynamic simulation we find that extended chemical groups can enhance nucleosome formation due to increased flexibility of the modified DNA duplex, and thus can serve as a new entry point for developing next-generation designer nucleosomes.

## MATERIALS AND METHODS

### DNA substrates

Details of DNA substrates used are given in Supplementary data, Materials and Methods. Briefly, linear DNA substrates for nucleosome assembly were constructed by PCR amplification from recombinant plasmid templates using Cy3- or Cy5-fluorescently labelled primers and Phusion DNA polymerase (NEB) (Supplementary Table S1). Following purification, with Qiaquick PCR purification kit (Qiagen) DNA concentration was determined using NanoDrop ND-1000 (Peqlab). Cy5-labeled DNA substrates were further used non-modified. Cy3-labeled substrates were either methylated or azidohexynylated using either wild-type M.SssI or M.HhaI methyltransferases together with a methyl group bearing cofactor SAM, or their engineered counterparts eM.SssI and eM.HhaI together with a synthetic cofactor analog bearing the azidohexynyl group, Ado-6-azide (17,19,24), respectively. For multiple methylations at CpG sites, M.SssI (Thermo Fisher Scientific) was used according to manufacturer's recommendations. For the multiple CpG azidohexynylations, 7 μg of DNA was incubated with 200 μM Ado-6-azide and 550 pmol eM.SssI (1:4 MTase:targets) in reaction buffer (10 mM Tris–HCl pH 7.4, 50 mM NaCl, 0.1 mg/ml BSA) at 30°C overnight. For methylation (mC) or azidohexynylation (ahyC) at only one site per substrate, 7 μg of DNA fragments were incubated with 200 μM *S*-adenosyl-l-methionine (SAM) or Ado-6-azide and 67 pmol eM.HhaI (1:1,5 MTase:targets) in reaction buffer (50 mM Tris–HCl pH 7.4, 0.5 mM EDTA, 0.2 mg/ml BSA) at 37°C overnight. DNA with 5-hydroxymethylated CpG sites was produced as described previously (25). Briefly, 10 μg of a DNA substrate was incubated with 5 μl M.SssI and 13 mM formaldehyde in a commercial M.SssI buffer at 25°C for 16 h, followed by Proteinase K digestion. The modified DNA was purified using DNA Clean & Concentrator kit (Zymo Research). Modification efficiency was determined by digesting the reaction products with modification-sensitive restriction endonucleases (Supplementary Figure S1). Substrates that showed full apparent protection from restriction nucleases were used for further experiments.

### Protein expression and purification

Calf thymus histones were purified from calf thymus essentially as described (26). Briefly, 100 mg nuclei pellet (kindly provided by G. Längst) was thawed and resuspended in 42 ml HAP 0.6 buffer (50 mM sodium phosphate pH 6.8, 600 mM NaCl) supplemented with protease inhibitors (100 μM PMSF, 20 μM Leupeptine and 14 μM Pepstatin) and 1 mM DTT. After sonication and centrifugation, the supernatant was incubated with 21 g equilibrated DNA Grade Bio-Gel HTP Hydroxyapatite (Bio-Rad) in batch at 4°C for 45 min. After four washing steps (centrifugation at 500 g for 2 min) with HAP 0.6 buffer, the histones were gravity-eluted using 10 ml of HAP 2.5 buffer (50 mM sodium phosphate pH 6.8, 2.5 M NaCl). Elution fractions were aliquoted, snap-frozen, and stored at −80°C or supplemented with glycerol and stored at − 20°C. Chromatin remodelers Snf2H and NoRC were expressed and purified from insect SF21 cells as described (27). SDS-PAGE images of purified histones and chromatin remodelers are shown in Supplementary Figure S2.

### Nucleosome assembly

Nucleosomes were assembled using the salt dialysis technique essentially as described previously (27). A typical assembly reaction (20 μl) contained 250 ng Cy3-labeled DNA (methylated or azidohexynylated) and 250 ng Cy5-labelled DNA (non-modified), 200 ng/μl BSA, 125 ng/μl competitor DNA and different amounts of calf thymus histone octamers. After dialysis 2μl of the assembly reaction was mixed with 8 μl Ex80 buffer (20 mM Tris-HCl pH 7.6, 1 mM MgCl_2_, 0.5 mM EGTA, 10% glycerol, 0.05% NP-50, 200 ng/μl BSA, 80 mM KCl) and with Purple Loading Dye without SDS (NEB). Samples were resolved on a 5% polyacrylamide native gel in 0.4× TBE. Gels were analyzed using Typhoon FLA-9500 (GE Healthcare) and MultiGauge v.3.0 (Fujifilm) software.

### Mapping of nucleosome positions

For restriction endonuclease mapping assays, restriction endonucleases RsaI and BmrI (NEB) were used. The reactions were carried out according to the manufacturer's recommendations and afterward the enzymes were heat-inactivated at 80°C for 20 min in the presence of SDS. Samples were resolved on 5% native polyacrylamide gels. Gels were stained with ethidium bromide and visualized using a gel imager (Intas).

For mapping nucleosome positions with MNase, 15 μl of readily assembled mononucleosomes on 80-Widom DNA template (non-modified or azidohexynylated) were supplemented with 1.7 μl 10 mg/mL BSA, 1 μl 3.5% NP-40 and 67.3 μl MNase buffer (20 mM Tris–HCl pH 7.6, 0.5 mM EGTA, 10% glycerol). The free DNA was digested by adding 21.3 μl of MNase master mix (632 mU MNase (Sigma-Aldrich), 20 mM CaCl_2_, 64 mM KCl, 16 mM Tris–HCl pH 7.6, 0.4 mM EGTA and 8% glycerol) and incubation at 37°C for 1 min. The reaction was stopped with 15.6 μl MNase stop buffer (3.3% SDS, 82 mM EDTA and 0.36 % glycogen) and placed on ice. To digest the histones, the reaction was supplemented with 1.2 μl Proteinase K (20 mg/ml, Sigma-Aldrich) and incubated at 50°C for 1 h, followed by heat inactivation at 95°C for 10 min. The undigested nucleosomal DNA was purified by phenol/chloroform/isoamyl alcohol extraction and ethanol precipitation. To produce blunt-ended inserts, the purified DNA was treated with T4 DNA Polymerase (NEB) in 1× CutSmart buffer, supplemented with 100 μM dNTPs. The reaction was stopped by adding EDTA to a final concentration of 10 mM and subsequent heat inactivation at 75°C for 20 min. Blunt-ended fragments were then cloned into the pCR-Blunt II-TOPO vector using the Zero Blunt PCR cloning kit (Life Technologies) and transformed into chemically competent XL1 blue cells. Positive clones were verified by colony PCR and sequenced. We aligned the resulting DNA sequences using the Genious program.

### Nucleosome stability assays

For thermal assays, 2 μl of nucleosome mixtures were mixed with 13 μl Ex80 buffer and incubated at different temperatures (25 (RT), 50.3, 58.9, 65.5 and 69.6°C for 80-Widom nucleosomes and 25 (RT), 50.6, 59.8, 65.0, 70.9 and 75.3°C for Widom and 36-Widom nucleosomes) for 2 h and 4 h, respectively. Afterward 3 μl of Purple Loading Dye without SDS (NEB) was added to the samples and samples were resolved on a 5% polyacrylamide native gel in 0.4× TBE. Gels were analyzed using Typhoon FLA-9500 (GE Healthcare) and MultiGauge v.3.0 (Fujifilm) software.

For ethidium bromide incorporation assays, 2 μl of nucleosome mixtures were added to 13 μl Ex80 buffer containing various ethidium bromide concentrations (up to 1 mM). Samples were incubated at 30°C for 30 min and further processed as described above.

### Nucleosome repositioning assay

For nucleosome repositioning assays, 2 μl of nucleosome mixtures were mixed with 100 nM Snf2H or NoRC in Ex100 buffer (20 mM Tris–HCl pH 7.6, 1 mM MgCl_2_, 0,5 mM EGTA, 10% glycerol, 100 mM KCl) supplemented with 1 mM ATP and reactions were incubated at 30°C for 45 min. The reactions were stopped by adding 250 ng competitor plasmid DNA. Afterward 3 μl of Purple Loading Dye without SDS 6× (NEB) was added and the samples were resolved on a 5% polyacrylamide native gel in 0.4× TBE. Gels were analyzed using Typhoon FLA-9500 (GE Healthcare) and MultiGauge v.3.0 (Fujifilm) software.

### Molecular dynamics simulations

A 25 base pairs (bp) DNA fragment 5′-GCTCTCTCGAAGCAACGAGAACAGT containing 2 CpG sites (underlined) in different sequence contexts, separated by 6 bp to avoid ahyC interaction between the two CpG sites, was selected for molecular dynamics (MD) analysis. 5-methyl or 5-(6-azidohex-2-ynyl) groups at CpG sites were added as shown in Figure 5A. The CHARMM-compatible topology for the modified ahyC nucleotide tail was built on a B-DNA structure based on azido and alkynyl group parameters optimized by Smith *et al.* (28). Molecular dynamics simulations were performed with GROMACS program (v. 2020.4) (29,30) using CHARMM36 force field (31) in water containing 0.15 M NaCl. Simulations were performed under constant temperature (293 K) and constant pressure (1 bar) in quintuplicate. DNA duplex simulations were run for 100 ns, while DNA–ethidium simulations were run for 1200 ns. DNA duplex trajectories were analyzed with the do_x3dna package (32), which uses the 3DNA (v.2.1) program (33). For more details, see Supplementary data, Materials and Methods. ANOVA single factor statistical analysis was carried out to evaluate the differences of the standard deviation of DNA parameters at base-pair steps no. 7–9 and 15–17 (i.e. including the base pairs no. 7–10 and 15–18) from the five MD replicas and Violin plots were generated using the Plotly (34) software.

## RESULTS

### ahyC modifications alter nucleosome positioning and affinity

**Figure 1.**
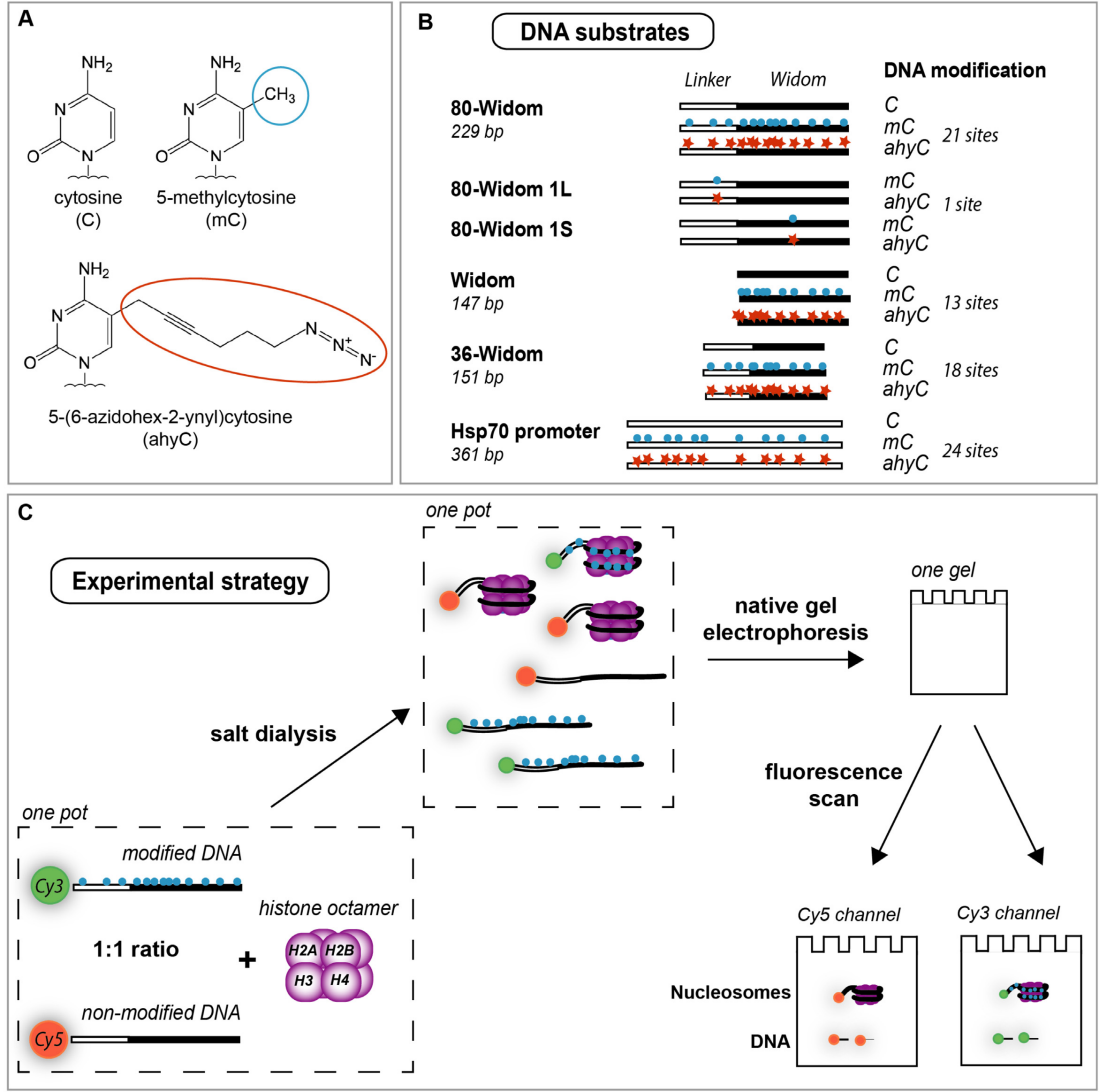
Schematic representation of the study. (**A**) DNA modifications examined in this study were 5-methylcytosine (mC) and 5-(6-azidohex-2-ynyl)cytosine (ahyC). (**B**) The panel of DNA substrates used for the nucleosome assembly assays. (**C**) Experimental strategy: DNA substrates (Cy3- and Cy5-labeled, modified and non-modified, respectively) were mixed with histone octamers in one pot and assembled into nucleosomes via salt dialysis. The assembly reactions were analyzed by native gel electrophoresis followed by fluorescence scan at both Cy5 and Cy3 fluorescence channels in parallel.

To investigate how a chemical extension of the 5-methyl group might affect the nucleosome assembly, positioning, and other properties of nucleosomes, we have generated a series of DNA substrates that were modified, with methyl, hydroxymethyl or azidohexynyl group, at the fifth position of the cytosine ring at CpG sites (Figure 1A, B, Supplementary Materials). The substrates included the Widom 601 sequence, which is known as a strong nucleosome positioning sequence (10), with or without any additional DNA linkers, as well as *Drosophila melanogaster* Hsp70 promoter sequence (35) (Figure 1B). To reliably compare the histone octamer binding to modified versus unmodified DNA *in vitro*, we used a one-pot approach whereby we mixed both the modified and unmodified substrates 5′-Cy3- and 5′-Cy5-labeled, respectively, in one sample at a 1:1 ratio together with calf thymus histone octamers and performed salt dialysis to assemble nucleosomes (Figure 1C). Histone bound and free DNA species were separated by native gel electrophoresis and visualized by separate scanning of the Cy3 and Cy5 fluorescence channels. This experimental setup allowed us to observe direct competition of the modified or non-modified substrates with respect to their nucleosome-building capacity.

**Figure 2.**
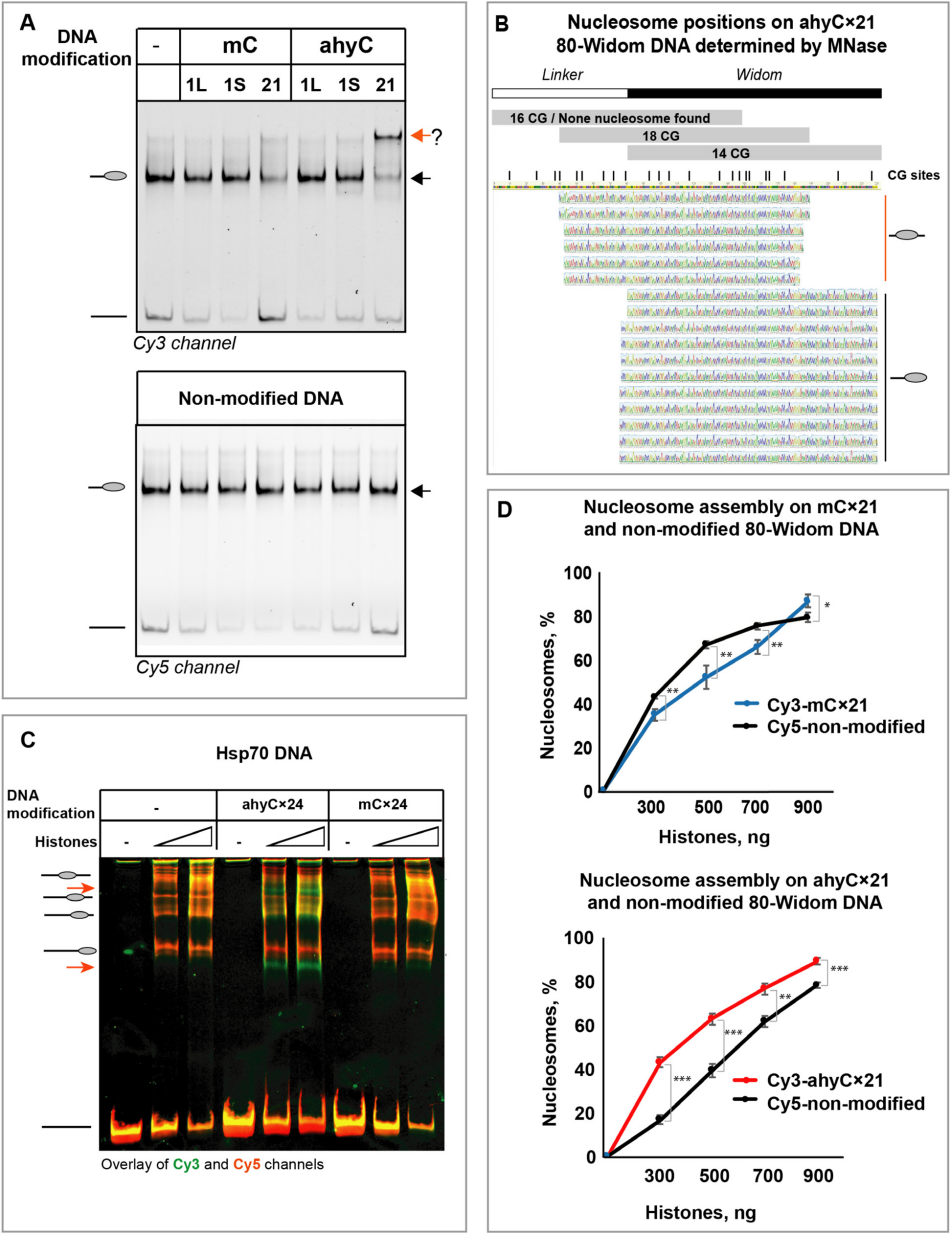
Multiple ahyC modifications of DNA changes nucleosome positioning and affinity. (**A**) EMSA of nucleosomes assembled on modified and non-modified 80-Widom DNA substrate. mC – the DNA substrate contains 5-methylcytosine, ahyC – 5-(6-azidohex-2-ynyl)cytosine. 1L – the modification is at one site in the linker sequence, 1S - the modification is at one site in the Widom sequence, 21 – the modification is introduced at 21 sites through all the substrate length. The black arrow points at nucleosome positioning characteristic to unmodified DNA, while the red arrow indicates the new nucleosome species formed with the ahyCx21 modified substrate. (**B**) Nucleosome positions on ahyCx21 80-Widom substrate. DNA was assembled into nucleosomes and subjected to MNase digestion. The nucleosome-protected fragments were cloned and sequenced. Blackline indicates the 3′-end nucleosome position on the Widom sequence, while the red line indicates a new nucleosome position formed at the center of the 80-Widom substrate. (**C**) Nucleosome assembly on Hsp70 promoter DNA sequence, either with multiple ahyC or mC modifications. Red arrows indicate new nucleosome positions formed in the presence of multiple ahyC modifications of the DNA substrate. The intensity traces are shown on the right side: in cyan is a trace for C, in purple – for mC and in orange - for ahyC. (**D**) Histone affinity to 80-Widom substrate, either non-modified (black) or multiply modified with mC (blue) or ahyC (red). Both modified and nonmodified DNA, 5′-Cy3- and 5′-Cy5-labelled, respectively, were mixed in the same pot at a 1:1 ratio with different concentrations of calf thymus histone octamers, and salt dialysis was applied to assemble the DNA into nucleosomes. Formed nucleosomes were further separated from free DNA by native gel electrophoresis. After visualization by both the Cy3 and Cy5 fluorescence channels, the percentage of nucleosome-bound DNA was determined. Data points are mean values with standard deviation from at least three independent experiments. Significance was calculated based on one-way ANOVA test, * *P*< 0.05, ** *P*< 0.01, *** *P*< 0.001.

Our first substrate was a DNA duplex comprising the well-known nucleosome positioning Widom 601 (further – Widom) sequence (147 bp) and an 80-mer linker of general DNA. The linker was generally expected to remain nucleosome-free but permit nucleosome repositioning if any exclusion effects arose due to the DNA modification (Figure 1B). We generated two one-site modification substrates: one in the linker region (11 bp away from Widom DNA) and the other in the Widom DNA sequence (74th bp in the Widom sequence), designated 80-Widom1L and 80-Widom1S, respectively. The nucleosomes formed on singly-modified DNAs always resulted in one slower migrating band in the gel, similar to those formed with the unmodified substrate (Figure 2A). This indicated that the octamer was bound at one DNA position, regardless of the modification location (linker or Widom DNA) or modification chemistry (mC or ahyC). To exclude the possibility that the nucleosome was positioned on the other end of the DNA duplex and thus exhibited similar electrophoretic behavior, we have performed a restriction digestion analysis of the assembled nucleosomes (Supplementary Figure S3). In all the unmodified and single-site-modified substrates, the Widom DNA sequence was protected from digestion, indicating that the nucleosome assembly occurred at the Widom DNA sequence and stayed robust despite the single-site modifications.

Therefore, we next turned to multiply-modified DNA substrates, which contained 21 modification sites (CpG) dispersed at naturally occurring variable intervals through all the DNA length (Figure 1B, Supplementary Materials). Both the multi-methylated DNA (mCx21) and non-modified DNA resulted in one shifted band in the EMSA experiments (Figure 2A) indicating that DNA methylation did not affect the nucleosome positioning. Remarkably, the ahyCx21 80-Widom DNA showed two shifted bands in EMSA, one of which (migrating faster) was at the same position as the nucleosomes assembled on the non-modified DNA (Figure 2A, black arrow), whereas the other (migrating slower) band (Figure 2A, red arrow) indicated the formation a new nucleosome species. Therefore we conclude that the nucleosomes were reconstituted on ahyCx21 80-Widom DNA with different positioning.

To determine the new nucleosome position, we conducted the restriction-digestion analysis which showed that the nucleosome-protected area relocated towards the center of the ahyC modified DNA substrate (Supplementary Figure S3). For high-resolution mapping, we carried out Micrococcal Nuclease footprinting experiments in which all but nucleosomal DNA is degraded. After cloning and sequencing of the remaining DNA fragments, we identified two nucleosome-protected areas (Figure 2B): in the middle of the DNA substrate (corresponding to the slowest migrating band in the EMSA, Figure 2A, red arrow) and on the Widom-end of the substrate DNA (corresponding to the same band in the EMSA as for unmodified DNA, Figure 2A, black arrow). Interestingly, the new nucleosome-protected region covers the majority (18/21) of the CpG sites present in the DNA substrate.

To exclude any possible sequence-dependent effects, we similarly examined another DNA substrate - the naturally occurring *D. melanogaster* Hsp70 DNA promoter sequence (35). The Hsp70 DNA fragment contains 24 cytosines at CpG sites that were enzymatically modified to produce mCx24 or ahyCx24 DNA. Since no strong DNA positioning sequence is present on this substrate, nucleosomes are distributed over several DNA sites and therefore are detected as multiple bands in EMSA experiments (Figure 2C). However, in comparison to the non-modified DNA, the nucleosomes assembled on ahyCx24 Hsp70 DNA produced two additional bands (Figure 2C, red arrows) on the EMSA, indicating different nucleosome positioning on the modified DNA. Altogether, our observations lend strong support for the conclusion that ahyC can alter the nucleosome positioning on DNA, and that this phenomenon is not sequence-specific.

### ahyC modifications recruit nucleosome

To determine how the DNA modifications affect the affinity for nucleosome formation, we measured the nucleosome assembly at a series of histone octamer concentrations. We observed almost identical DNA incorporation into nucleosomes when non-modified and mono-modified substrates were tested (Supplementary Figure S4) indicating that cytosine modifications at a single site are insufficient to alter nucleosome assembly. However, the mCx21 Widom-80 DNA showed weaker binding of the octamer than the unmodified reference DNA present in the same reaction (Figure 2D, upper panel and Supplementary Figure S4D). In contrast, the ahyCx21 Widom-80 DNA was preferred for assembly in a mixture with the non-modified DNA (Figure 2D, lower panel and Supplementary Figure S4D). Furthermore, the hydroxymethylation of the 80-Widom DNA did not affect nucleosome positioning and affinity (Supplementary Figure S4E). This confirms previous findings of weaker interactions of methylated DNA with the histone octamer and indicates a favorable effect of the extended cytosine-5 modifications on nucleosome formation.

To avoid any ambiguity deriving from nucleosome repositioning, we similarly examined two shorter DNA substrates that were just long enough to form a nucleosome and thus precluded any nucleosome repositioning. The substrates represent (i) the Widom sequence (147 bp), and (ii) a fragment of 80-Widom that maps to the new nucleosome position provoked by the ahyC modifications, according to MNase digestion results (151 bp) (Figure 2B). The latter substrate, designated 36-Widom, bears a part (36 bp) of the linker DNA and a part of the Widom sequence (Figure 1B). Widom DNA carries 13 CpG sites and 36-Widom DNA has 18 CpG sites that can be modified (Figure 2B).

**Figure 3.**
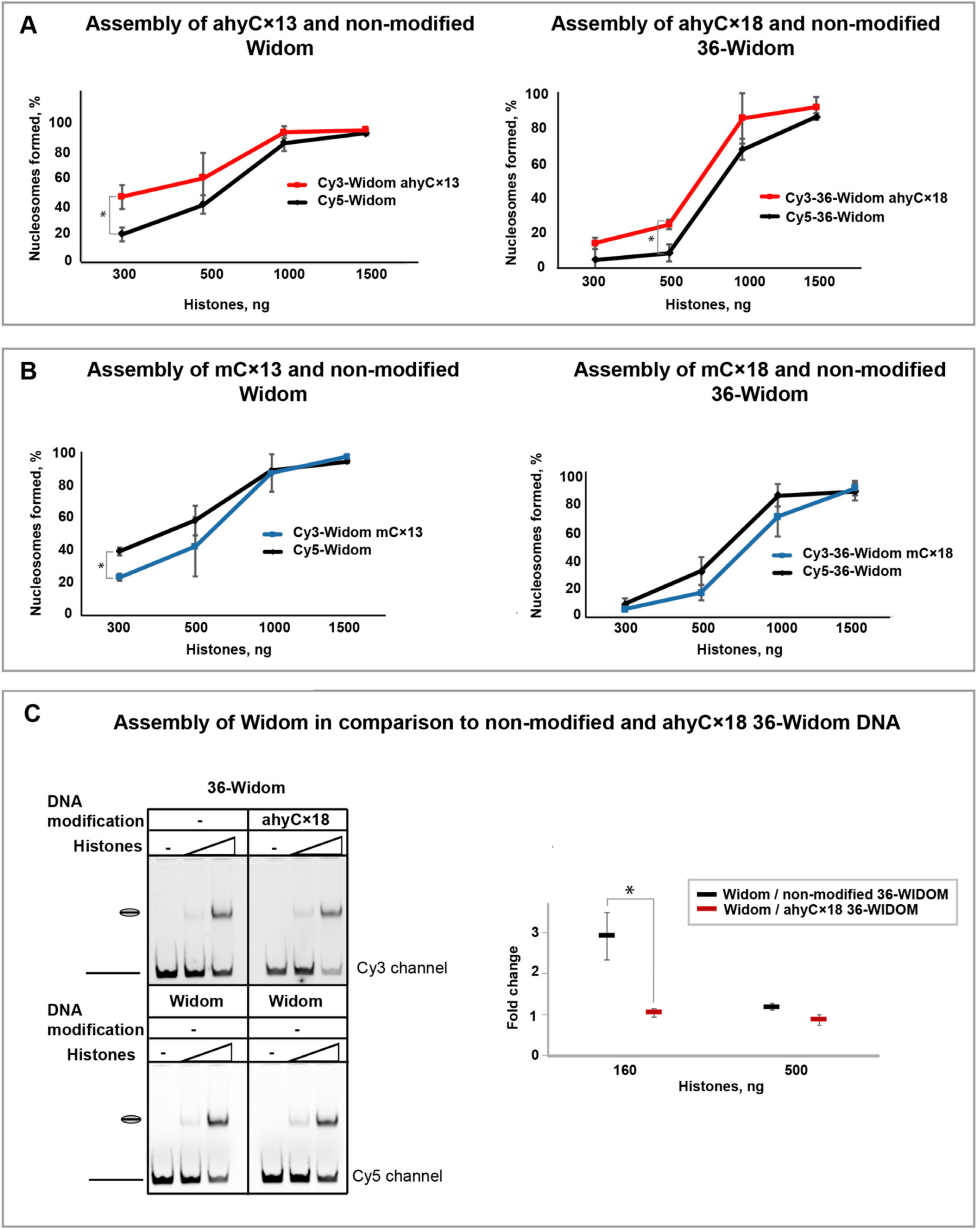
The interplay between the DNA sequence and multiple ahyC modifications in the determination of the histone affinity. (**A**) Histone affinity to Widom (left panel) and 36-Widom (right panel) substrates, either non-modified (black) or multiply modified with ahyC (red). Both modified and nonmodified DNA, 5′-Cy3- and 5′-Cy5-labeled, respectively, were mixed in the same sample at a 1:1 ratio together with different concentrations of calf thymus histone octamers. Salt dialysis was applied to assemble nucleosomes which were further separated from free DNA by native gel electrophoresis. After visualization by both the Cy3 and Cy5 fluorescence channels, the percentage of nucleosome-bound DNA was determined separately for the modified and non-modified substrates. (**B**) Histone affinity to Widom (left panel) and 36-Widom (right panel) substrates, either non-modified (black) or multiply modified with mC (blue). (**C**) Multiply ahyC modification of 36-Widom makes the substrate as affine to histones as the well-known nucleosome assembly sequence Widom. Non-modified Widom substrate was mixed with either non-modified or multiply ahyC modified 36-Widom together with different histone concentrations. The nucleosome assembly was evaluated after native gel electrophoresis (left panel). The difference in which non-modified Widom DNA substrate was preferably assembled into nucleosomes, and the loss of preferences after 36-Widom modification, are plotted in the right panel. Data points are mean values with standard deviation from at least three (A and B) or two (C) independent experiments. Significance was calculated based on one-way ANOVA or t-test (two sample, equal variance), * *P*< 0.05.

Using the new substrates modified at the CpG sites, we performed the nucleosome affinity assay as described above. Remarkably, the ahyCx13 Widom DNA was preferably incorporated into nucleosomes as compared to the non-modified Widom (Figure 3A, left panel) indicating, that ahyC does not inhibit the nucleosome formation on the Widom DNA sequence but rather promotes it further. Similarly, ahyCx18 36-Widom assembled into nucleosomes with a higher affinity than the unmodified control (Figure 3A, right panel). In contrast, and in line with the 80-Widom DNA experiments, the multi-site CpG methylation resulted in a somewhat lower affinity for both short substrates (Figure 3B). Therefore, we conclude that ahyC modifications reposition the nucleosome on the 80-Widom substrate due to the emergence of a new strong nucleosome positioning site, rather than its eviction from the ahyC-modified Widom sequence.

Finally, to elucidate the interplay between the sequence and multiple ahyC modification-driven nucleosome positioning, we analyzed the nucleosome assembly with Widom and 36-Widom substrates in non-modified or multiple ahyC-modified states. We observed that Widom DNA was the preferred substrate for nucleosome formation in comparison to the non-modified 36-Widom. In the presence of lower histone concentrations, there were 3 fold more nucleosomes wrapped on the Widom than on 36-Widom (Figure 3C). However, both the Widom and ahyCx18 36-Widom DNA substrates assembled into nucleosomes equally well at both histone concentrations examined. This is in line with our MNase digestion analysis, where the ahyC modification resulted in nucleosome-protected areas at both the Widom sequence and the 36-Widom region. This observation demonstrates that ahyC modifications can override the sequence-driven preference (such as Widom sequence) by creating equally attractive nucleosome binding sites in DNA.

### ahyC affects nucleosome stability

**Figure 4.**
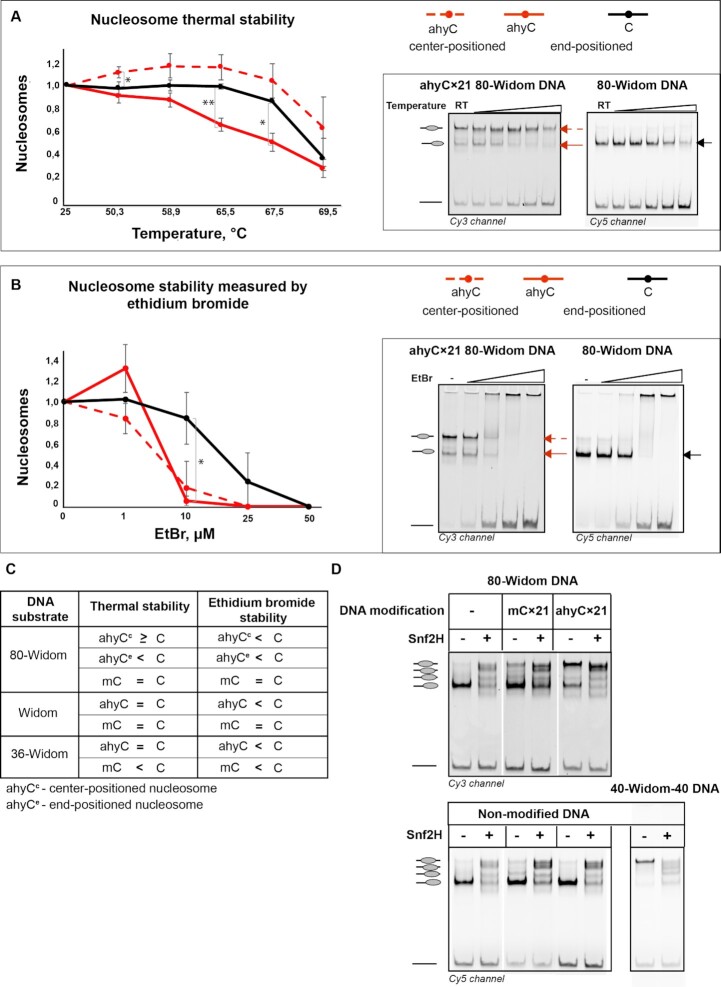
Multiple ahyC DNA modification affects nucleosome stability and repositioning by chromatin remodeler Snf2H. (**A**) Thermal stability of nucleosomes assembled in one pot on both non-modified and multiply ahyC modified 80-Widom substrate. Data points are mean values with standard deviation from at least three independent experiments. Significance was calculated based on one-way ANOVA, * *P*< 0.05, ** *P*< 0.01. (**B**) Disruption of nucleosomes by intercalation of ethidium bromide, in one pot of mixed, both non-modified and multiply ahyC modified 80-Widom substrate. Data points are mean values with standard deviation from at least three independent experiments. Significance was calculated based on one-way ANOVA, * *P*< 0.05. (**C**) Summary of nucleosome stability on different substrates, modification states, and thermal or ethidium bromide challenging. (**D**) Nucleosome repositioning by chromatin remodeler Snf2H. Nucleosomes were assembled in one pot on both non-modified and modified 80-Widom substrate (mCx21 or ahyCx21) or separately on non-modified 40-Widom-40 substrate. Assembled nucleosomes were then incubated with Snf2H and resolved by native gel electrophoresis.

To obtain further insights into the properties of the ahyC-promoted nucleosome stability, we monitored the temperature-induced DNA release from the nucleosome using EMSA (Figure 4A). With 80-Widom DNA, we observed a clear weakening of the higher migrating band and increasing the lower (free DNA) band intensities, indicating a gradual temperature-induced release of DNA from the nucleosomes. The nucleosome assembled on ahyCx21 80-Widom DNA substrate and migrating higher (center-positioned nucleosome, red dashed arrow) displayed similar to slightly higher thermal stability compared to the non-modified DNA (Figure 4A, C), whereas the lower migrating (the end-positioned nucleosome, red arrow) vanishes, which could be interpreted as temperature-induced nucleosome sliding towards the ahyC-modified sites. Temperature-induced nucleosome redistribution on unmodified DNA was reported before (36). Nucleosomes assembled on methylated (Supplementary Figure S5A), as well as short Widom and 36-Widom modified DNAs displayed similar thermal stabilities (Figure 4C, Supplementary Figures S6 and S7) suggesting that the DNA modifications do not strongly affect the nucleosome thermal stability.

To further examine the robustness of the histone-DNA interactions to conformational perturbations of the bound DNA, we probed the reconstituted nucleosomes with increasing concentrations of ethidium bromide and monitored the release of free DNA by EMSA. Ethidium bromide is a DNA intercalating molecule that has nucleosome destabilizing properties (37). Unexpectedly, with nucleosomes reconstituted on non-modified and ahyCx21 80-Widom DNA, we found that the latter was disrupted at lower concentrations of ethidium bromide (Figure 4B, C), indicating lower stability of the ahyC-modified nucleosomes. With the both short DNA substrates, similar results were obtained (Figure 4C and Supplementary Figures S8 and S9), while methylation did not significantly enhance the nucleosome susceptibility to ethidium bromide (Supplementary Figures S5B, S8C, S9C). Therefore we concluded that nucleosomes assembled on ahyC-modified DNA are more sensitive to ethidium bromide presumably because ethidium bromide can easier intercalate in azidohexynylated DNA. To test this notion, we performed fluorescent measurement of the free ahyC-DNA interaction with ethidium bromide and found no significant difference between the multiply ahyC-modified and unmodified DNA (Supplementary Figure S10A). We further hypothesized that DNA bending in nucleosomes may also play a role in facilitating ethidium bromide intercalation, and our molecular dynamics simulation experiment lends support to this idea (Supplementary Figure S10B, C). Yet the mechanism of this synergistic effect of ahyC modification and DNA bending remains to be determined.

### ahyC nucleosomes repositioned by chromatin remodelers

Finally, we examined if the action of chromatin remodelers could be affected by the ahyC modifications on DNA. Snf2H is a catalytic subunit of several chromatin remodeler complexes and can reposition nucleosomes *in vitro* on its own or in a complex with non-catalytic subunit Tip5 (chromatin remodeling complex NoRC). First, we tested whether Snf2H could reposition the nucleosomes on modified 80-Widom DNA. As expected, Snf2H repositions the end-positioned nucleosomes on non-modified 80-Widom and center-positioned nucleosomes on 40-Widom-40 DNA to several other positions (Figure 4D). Nucleosomes assembled on singularly-modified DNA containing mC or ahyC at one site or multiply methylated mCx21 DNA were repositioned by Snf2H and NoRC similarly as nucleosomes on non-modified DNA (Supplementary Figure S5C,D). However, nucleosomes assembled on ahyCx21 80-Widom DNA were remodeled differently (Figures 4D and S5D). Taken together these data indicate that the chromatin remodelers Snf2H and NoRC reposition the assembled nucleosomes despite the cytosine modification chemistry or the number of modified sites on the DNA molecule. However, ahyC present at multiple DNA sites affects the final nucleosome positioning in the Snf2H remodeling reaction.

### ahyC promotes local structural fluctuations in the DNA helix

**Figure 5.**
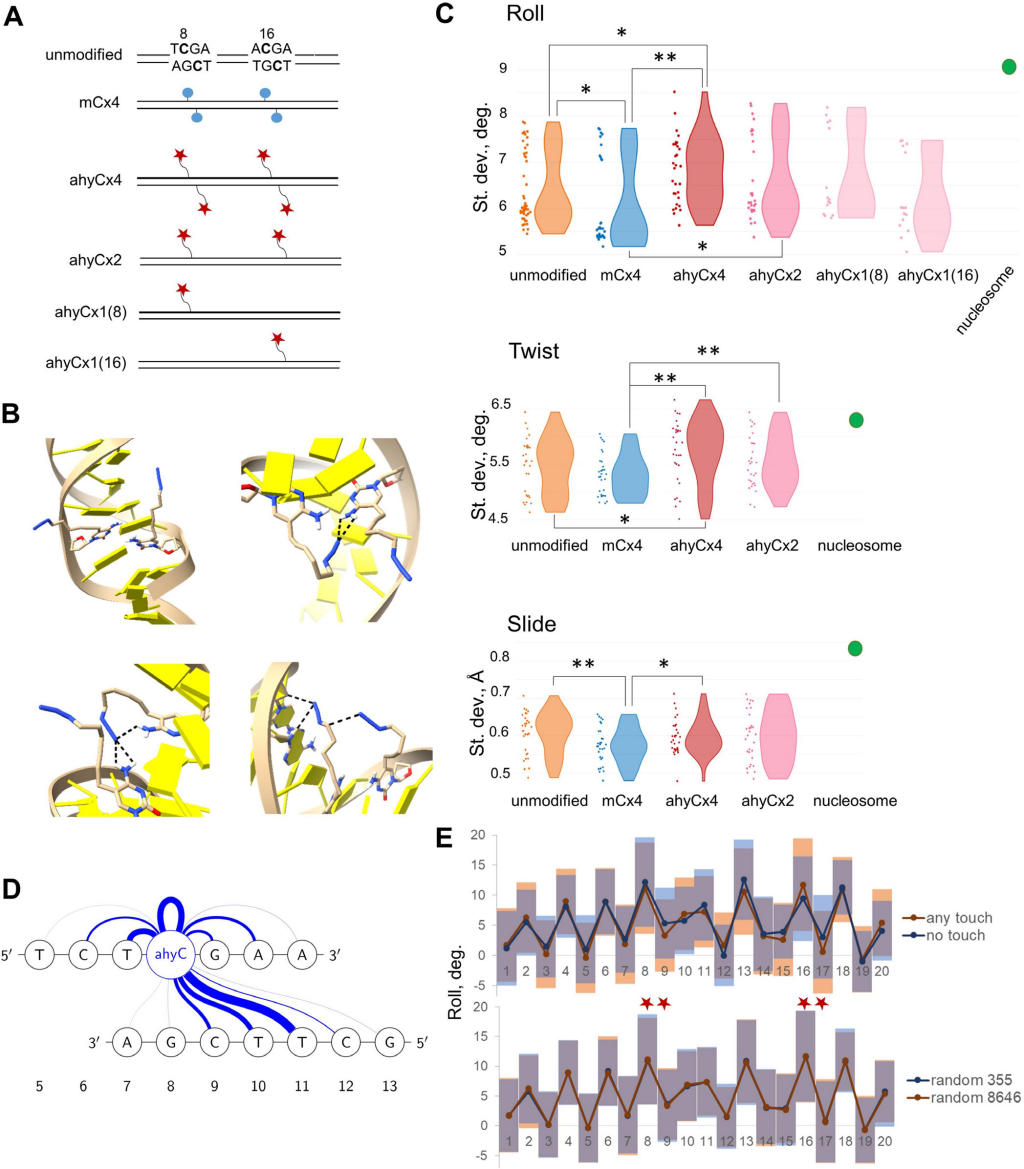
The structural fluctuations of ahyC-modified DNA are revealed by molecular dynamics simulations. (**A**) 25-bp long DNA fragments, modified at CpG sites and used for molecular dynamics simulations. The CpG sites and the two adjacent base pairs are outlined, the modified C is in bold. Blue dot – mC, red star – ahyC. (**B**) Varied ahyC conformations and a few examples of interactions in the ahyCx4 fragment observed during the molecular dynamics simulation. (**C**) The standard deviation of base-pair step parameters Roll, Twist, and Slide at modified sites (the CpG and one adjacent base pair on each side). Five independent molecular dynamics simulations were run for each DNA fragment. The resulting DNA parameters were analyzed using 3DNA software. The SD at the base pair steps 7–9 and 15–17 (encompassing the base pairs 7–10 and 15–18, where no. 8 and 16 are modified, plus no. 9 and 17 modified at the other strand in fully modified fragments) is plotted. ANOVA analysis showed statistically reliable differences between the analyzed fragments, * *P*< 0.1, ** *P*< 0.05. The respective parameter SD were also calculated throughout the DNA length in the nucleosome structure (PDB ID 3LZ0) – shown as a single green dot. (**D**) Schematic representation of dynamic ahyC group interactions with neighbor sites. The loop at ahyC itself represents the ahy group azide interaction with the C base. The blue line thickness corresponds to the cumulative frequency at which the interaction occurs during the time of MD simulation. The time points of MD trajectories (five independent simulations with 9001 time points each) of the ahyCx1(8) substrate were sorted in regard of the azide group being close (less than 4.5 Å) to DNA and the interaction site. Please see supplementary Table S2 for details. (**E**) Azide group-DNA interactions induce dinamic conformational changes in DNA as manifested by shifted Roll mean values around the modified sites and general shift in SD values, encompassing the accepted Roll conformations, throughout the DNA fragment length. 9001 time points of ahyCx4 molecular dynamics simulation trajectory were sorted into two groups: the ones with all the four ahyC azide groups exposed to the solvent (more than 4.5 Å away from DNA) – ‘no touch’ (355 points); and the ones that had at least one ahyC azide group close to DNA (less than 4.5 Å) – ‘any touch’ (8646 points) – upper panel. The same starting set of time points was randomly divided into two groups of the same size – lower panel. Mean Roll values with SD at every base pair step are shown. ahyC modifications are marked with asterisks.

Nucleosome formation confers substantial bending of the DNA helix, which is strongly dependent on the DNA flexibility (38–42). There is mounting evidence that natural cytosine modifications affect the DNA flexibility in different ways (mC and 6mA reduce, whereas hmC and fC enhance the flexibility), consequently affecting nucleosome assembly (11,43–47). We therefore hypothesized that the observed favorable ahyC effects on nucleosome formation could derive from ahyC modification-induced increase in DNA flexibility. To test this hypothesis we carried out molecular dynamics (MD) simulations of a model 25-bp DNA fragment containing two CpG sites separated by six base pairs, located in different sequence contexts and having several variations of the mC and ahyC modification patterns (Figure 5A). The 3DNA program was used to characterize the DNA conformations in terms of inter-base-pair (roll, tilt, twist, slide, shift, and rise) and intra-base-pair (propeller) structural parameters, which are relevant to describing the DNA curvature during the nucleosome assembly (39). During five independent 100 ns MD simulations, we observed conformational motions of the ahyC groups in the major groove as well as their interactions with adjacent DNA sites (Figure 5B, Supplementary Movie S1). At the same time, the base-pair parameter analysis showed increased variations of the roll, slide and twist parameters at the ahyC modified sites as manifested by higher standard deviation values (SD) compared to the unmodified and methylated DNA (Figure 5C). In general, a broader conformational distribution points at a higher flexibility of the molecule. Moreover, roll, slide, and twist are described as the most important contributors to the change of DNA curvature during the nucleosome assembly (39). The CpG and the two adjacent base pairs, one at each side, were the most significantly affected (Figure 5A, C, Supplementary Figure S11). In contrast, the mCx4 fragment showed lower SDs of the corresponding parameters, in agreement with an independently reported enhanced stiffness of methylated DNA (11). Compared to mCx4, the roll and twist parameters displayed more fluctuations even on ahyCx2 (hemi-modified) substrate (Figure 5C). Interestingly, the two mono-substituted substrates, ahyCx1(8) and ahyCx1(16), showed opposing SD distributions with respect to the unmodified control, indicating that the effects of individual modifications imbedded in different sequence contexts may not be well predictable (Figure 5C, Supplementary Figure S11A). To this end, the multiply modified fragments, ahyCx4 and ahyCx2, appear to be better representatives of the DNA substrates that were observed to promote nucleosome assembly.

Interestingly, other parameters such as shift, tilt, and rise, are more stable in both methylated and ahyC DNA as opposed to unmodified control (Supplementary Figure S11A). This can be explained by steric hindrance and enhanced stacking. The propeller showed no major differences which seems to largely depend on the sequence (base-pairing) context rather than modifications in the major groove. Additionally, both cytosine modifications, mC and ahyC significantly widen the major groove (Supplementary Figure S12). Altogether, we conclude that the increased variations of the roll, slide and twist parameters at the ahyC modified sites reflect increased flexibility of DNA substrates and a lower energetic barrier for the histones to form a nucleosome. Yet, the possibility that the ahyC groups directly interact with the histones and thereby enhance the nucleosome formation can not be excluded by our experiments. Even though the ahyC groups are rather long (up to 12 Å) (Supplementary Figure S13), they contain no negatively charged or hydrophobic groups that could be expected to make strong bonding with the histones. Thus, we believe that the enhanced flexibility of ahyC DNA is the main cause of the directed nucleosome assembly, and the histone-ahyC group interactions may be an additional factor.

### DNA flexibility is promoted by dynamic interactions of ahyC groups with adjacent nucleotides

To gain further insights into the mechanism of how the ahyC promotes the DNA structural plasticity and enhances its flexibility, we looked at possible interactions of the ahy groups with adjacent nucleotides in the fragment (Figure 5D, Supplementary Table S2). Preliminary inspection of the DNA structures in the MD trajectory showed that the ahyC groups were often bent inwards with their terminal azide group coming into proximity with other DNA bases, backbone atoms, and adjacent ahyC groups. The azide group contacts with the neighbouring DNA residues were typically short-lived (∼0.1 ns). However, these motions were accompanied by large conformational motions of the DNA including DNA bending along the helical axis. We therefore went on to determine if these dynamic side chain interactions indeed correlate with the global changes in DNA structure thereby rendering additional flexibility. For this, we sorted 9001 time points of the ahyCx4 molecular dynamics trajectory to those with at least one of the four ahyC azide group (the terminal N atom) located <4.5 Å away from any DNA atom (‘any touch’ group) and those with all four ahyC azide groups pointing into solvent (>4.5 Å from the DNA, ‘no touch’ group). It turned out that ∼95% of the time points (Supplementary Table S3) fell in the ‘any touch’ group highlighting the prevalence of intramolecular non-covalent interactions between the tethered azide and the rest of DNA. The ‘any touch’ group showed broadened and shifted distributions of the roll (Figure 5E upper panel), twist, and slide (Supplementary Figure S14) as compared to the ‘no touch’ group. To exclude the possibility that the observed differences derived from artificial sorting of the time points, we randomly assigned the time points into two groups of identical sizes and found no detectable differences of the helical parameters between such groups (Figure 5E lower panel and Supplementary Figure S14). This supports the idea that local interactions of the azidohexynyl group promote dynamic structural deviations of the DNA helix itself. Inspection of individual examples of interaction sites and structural snapshots of MD time points illustrate how ahyC–DNA interactions invoke alterations of the helical parameters (roll and twist) around the interacting modified base (Supplemental Figures S15 and S16A). We also witnessed occasional more complicated contacts, involving both ahyC groups at the CpG sites interacting with each other and/or neighbor bases (Supplementary Figure S16B), which we did not analyze in detail but believe they also contribute to the overall DNA flexibility effect.

Altogether our observations suggest that the azidohexynyl side chains enhance DNA flexibility by making dynamic favorable interactions with neighboring DNA sites thereby broadening the range of accepted helical conformations that are less frequent in unmodified and methylated DNA.

## DISCUSSION

It is suggested that DNA modifications that alter its physical properties will most likely affect the assembly of nucleosomes (40,41). Indeed, CpG and non-CpG methylation affect DNA micromechanical properties, i.e. methylation adds stiffness to DNA (11,44) and therefore reduces the affinity of the DNA to assemble into nucleosomes (43,45). Additionally, it might affect the nucleosome positioning as it was shown for satellite 2 region in pericentric heterochromatin domain, whereas the nucleosome structures and thermal stability stays unaffected (46). Furthermore, successive oxidation of 5mC by TET family enzymes yields other modifications of cytosine, where 5hmC and 5fC (5-formylcytosine) enhance the DNA flexibility, and 5-caC (5-carboxylcytosine) does not have a measurable effect (11), and it was reported to be related to nucleosome formation (15). Altogether, there is an intimate interplay between the DNA features affected by cytosine modification at fifth position and nucleosome assembly as well as positioning. However, to our knowledge, synthetic DNA modifications have not been studied in nucleosome assembly nor characterized in terms of DNA physical properties yet.

Thus, in this study, we for the first time examined the plasticity of mononucleosomes in regard to extended bioorthogonal cytosine modification at CpG sites. Unexpectedly we found striking effects of azidohexynylated DNA substrates on the formation as well as the properties of the assembled nucleosomes. Modifications of cytosine at the fifth position point to the major groove of B-DNA, while histone interactions occur to the backbone of DNA or the minor groove. Thus direct either positive or negative interactions with histones that would affect nucleosome formation are unlikely. Even though the ahyC groups are rather long and flexible enough to occasionally get close to the histone part of a nucleosome, we believe the impact of these interactions is minor (Supplementary Figure S13). Multiple-site methylated DNA was reproducibly wrapped into nucleosomes with lower affinity compared to the non-modified DNA, in accordance with the more open nucleosome structure on methylated DNA reported before (47). In contrast, azidohexynylated DNA substrates were reconstituted into nucleosomes with a higher affinity compared to the non-modified DNA. Surprisingly, some nucleosomes were even positioned differently on the long multiple ahyC modified DNA substrates (ahyCx21 80-Widom and ahyCx24 Hsp70), preferably at the most densely modified region. Hence, based on our nucleosome assembly experiments, performed on various DNA substrates, we conclude that the long azidohexynyl group present at multiple CpG sites do not inhibit nucleosome assembly, but rather promotes it. MD simulations of ahyC-modified DNA revealed details of how ahyC groups dynamically interact with neighbouring nucleotides promoting structural fluctuations at proximal DNA sites. This, in turn, translates into a higher DNA flexibility, bendability, and ultimately, nucleosome preference for the multiply azidohexynylated DNA regions, as DNA flexibility has been shown to be a critical factor in nucleosome dynamics and mechanical stability (42). Further, ahyC DNA nucleosomes can be repositioned by Snf2H chromatin remodeler and display similar thermal stability, indicating that ahyC nucleosomes share similar properties with natural nucleosomes. On the other hand, ahyC modification renders lower stability of the same nucleosomes when exposed to the intercalator ethidium bromide. Altogether, our data for the first time show that enzymatically introduced non-natural cytosine analogs in DNA substrates are compatible with nucleosome formation, and may affect nucleosome stability, and repositioning by chromatin remodeling proteins.

Our findings open new doors to synthetic epigenetics by demonstrating that nucleosome formation could be directed to predetermined loci on DNA without changes of the genetic sequence itself. Such designer nucleosomes with either natural or synthetic DNA modifications could be used as model objects in epigenetic research and employed in biotechnology, synthetic biology, nanostructure assembly, etc. To this end, our study raises many questions for future study such as, how many ahyC modifications at a particular DNA site are required to recruit the nucleosome, or if we first form the nucleosome, and then deposit azidohexynylate, can nucleosome slide towards ahyC DNA, similarly as it does in our thermal stability assays (Figure 4A). Theoretically, it is hard to imagine that a single modification (or nucleotide change) in a 150 nt long stretch of DNA be sufficient to confer a significant effect on the nucleosome formation. The nucleosome contains several sharp bends of the DNA helix, and an appreciable effect is more likely to be achieved if the majority of the bends are supported by at least one ahyC modification, consistent with our observations. Furthermore, it would be interesting to probe even more bulky, aromatic cytosine modifications to find the limit of steric tolerance. Apart from nucleosome research, the ability to site-specifically modify DNA with synthetic groups that affect DNA flexibility, and also can be used for labeling or conjugation (such as ahy), may empower new discoveries in the research areas of other DNA-interacting proteins, nanostructure design, etc. All in all, as the azidohexynyl group is now getting extensively used for new applications in different DNA and RNA studies, it is of great importance to know in detail its effects on biochemical and biophysical properties of DNA.

## Supplementary Material

gkac444_Supplemental_Files

## References

[1] Luger K. , Mäder A.W., Richmond R.K., Sargent D.F., Richmond T.J. Crystal structure of the nucleosome core particle at 2.8 Å resolution. Nature. 1997; 389:251–260. 9305837 10.1038/38444

[2] Kornberg R.D. , Lorch Y. Primary role of the nucleosome. Mol. Cell. 2020; 79:371–375. 32763226 10.1016/j.molcel.2020.07.020

[3] Khorasanizadeh S. The nucleosome: from genomic organization to genomic regulation. Cell. 2004; 116:259–272. 14744436 10.1016/s0092-8674(04)00044-3

[4] Schrader A. , Gross T., Thalhammer V., Längst G. Characterization of dnmt1 binding and DNA methylation on nucleosomes and nucleosomal arrays. PLoS One. 2015; 10:e0140076. 26496704 10.1371/journal.pone.0140076 PMC4619679

[5] Felle M. , Hoffmeister H., Rothammer J., Fuchs A., Exler J.H., Längst G. Nucleosomes protect DNA from DNA methylation in vivo and in vitro. Nucleic Acids Res. 2011; 39:6956–6969. 21622955 10.1093/nar/gkr263 PMC3167622

[6] Becker P.B. , Workman J.L. Nucleosome remodeling and epigenetics. Cold Spring Harb. Perspect. Biol. 2013; 5:a017905. 24003213 10.1101/cshperspect.a017905 PMC3753709

[7] Längst G. , Manelyte L. Chromatin remodelers: from function to dysfunction. Genes (Basel). 2015; 6:299–324. 26075616 10.3390/genes6020299 PMC4488666

[8] Oppikofer M. , Bai T., Gan Y., Haley B., Liu P., Sandoval W., Ciferri C., Cochran A.G. Expansion of the ISWI chromatin remodeler family with new active complexes. EMBO Rep. 2017; 18:1697–1706. 28801535 10.15252/embr.201744011 PMC5623870

[9] Richmond T.J. , Davey C.A. The structure of DNA in the nucleosome core. Nature. 2003; 423:145–150. 12736678 10.1038/nature01595

[10] Lowary P.T. , Widom J. New DNA sequence rules for high affinity binding to histone octamer and sequence-directed nucleosome positioning. J. Mol. Biol. 1998; 276:19–42. 9514715 10.1006/jmbi.1997.1494

[11] Ngo T.T.M. , Yoo J., Dai Q., Zhang Q., He C., Aksimentiev A., Ha T. Effects of cytosine modifications on DNA flexibility and nucleosome mechanical stability. Nat. Commun. 2016; 7:10813. 26905257 10.1038/ncomms10813 PMC4770088

[12] Ehrlich M. , Gama-Sosa M.A., Huang L.H., Midgett R.M., Kuo K.C., McCune R.A., Gehrke C. Amount and distribution of 5-methylcytosine in human DNA from different types of tissues of cells. Nucleic Acids Res. 1982; 10:2709–2721. 7079182 10.1093/nar/10.8.2709 PMC320645

[13] Collings C.K. , Anderson J.N. Links between DNA methylation and nucleosome occupancy in the human genome. Epigenetics Chromatin. 2017; 10:18. 28413449 10.1186/s13072-017-0125-5 PMC5387343

[14] Portela A. , Liz J., Nogales V., Setién F., Villanueva A., Esteller M. DNA methylation determines nucleosome occupancy in the 5′-CpG islands of tumor suppressor genes. Oncogene. 2013; 32:5421–5428. 23686312 10.1038/onc.2013.162 PMC3898323

[15] Teif V.B. , Beshnova D.A., Vainshtein Y., Marth C., Mallm J.-P., Höfer T., Rippe K. Nucleosome repositioning links DNA (de)methylation and differential CTCF binding during stem cell development. Genome Res. 2014; 24:1285–1295. 24812327 10.1101/gr.164418.113 PMC4120082

[16] Nadal S. , Raj R., Mohammed S., Davis B.G. Synthetic post-translational modification of histones. Curr. Opin. Chem. Biol. 2018; 45:35–47. 29501025 10.1016/j.cbpa.2018.02.004

[17] Lukinavičius G. , Lapinaitė A., Urbanavičiūtė G., Gerasimaitė R., Klimašauskas S. Engineering the DNA cytosine-5 methyltransferase reaction for sequence-specific labeling of DNA. Nucleic Acids Res. 2012; 40:11594–11602. 23042683 10.1093/nar/gks914 PMC3526304

[18] Kriukienė E. , Labrie V., Khare T., Urbanavičiūtė G., Lapinaitė A., Koncevičius K., Li D., Wang T., Pai S., Ptak C. et al. DNA unmethylome profiling by covalent capture of CpGsites. Nat. Commun. 2013; 4:2190. 23877302 10.1038/ncomms3190

[19] Staševskij Z. , Gibas P., Gordevičius J., Kriukienė E., Klimašauskas S. Tethered oligonucleotide-primed sequencing, TOP-Seq: a high-resolution economical approach for DNA epigenome profiling. Mol. Cell. 2017; 65:554–564. 28111014 10.1016/j.molcel.2016.12.012 PMC5291818

[20] Wand N.O. , Smith D.A., Wilkinson A.A., Rushton A.E., Busby S.J.W., Styles I.B., Neely R.K. DNA barcodes for rapid, whole genome, single-molecule analyses. Nucleic Acids Res. 2019; 47:e68. 30918971 10.1093/nar/gkz212 PMC6614835

[21] Tomkuvienė M. , Ikasalaitė D., Slyvka A., Rukšėnaitė A., Ravichandran M., Jurkowski T.P., Bochtler M., Klimašauskas S. Enzymatic hydroxylation and excision of extended 5-methylcytosine analogues. J. Mol. Biol. 2020; 432:6157–6167. 33065111 10.1016/j.jmb.2020.10.011 PMC7763475

[22] Gordevičius J. , Narmontė M., Gibas P., Kvederavičiūtė K., Tomkutė V., Paluoja P., Krjutškov K., Salumets A., Kriukienė E. Identification of fetal unmodified and 5-hydroxymethylated CG sites in maternal cell-free DNA for non-invasive prenatal testing. Clin. Epigenetics. 2020; 12:153. 33081811 10.1186/s13148-020-00938-x PMC7574562

[23] Heck C. , Torchinsky D., Nifker G., Gularek F., Michaeli Y., Weinhold E., Ebenstein Y. Label as you fold: methyltransferase-assisted functionalization of DNA nanostructures. Nanoscale. 2020; 12:20287–20291. 33001091 10.1039/d0nr03694c

[24] Lukinavičius G. , Tomkuvienė M., Masevičius V., Klimašauskas S. Enhanced chemical stability of adomet analogues for improved methyltransferase-directed labeling of DNA. ACS Chem. Biol. 2013; 8:1134–1139. 23557731 10.1021/cb300669x

[25] Liutkevičiūtė Z. , Lukinavičius G., Masevičius V., Daujotytė D., Klimašauskas S. Cytosine-5-methyltransferases add aldehydes to DNA. Nat. Chem. Biol. 2009; 5:400–402. 19430486 10.1038/nchembio.172

[26] Laybourn P.J. , Kadonaga J.T. Role of nucleosomal cores and histone H1 in regulation of transcription by RNA polymerase iI. Science. 1991; 254:238–245. 1718039 10.1126/science.254.5029.238

[27] Manelyte L. , Strohner R., Gross T., Längst G. Chromatin targeting signals, nucleosome positioning mechanism and non-coding RNA-mediated regulation of the chromatin remodeling complex NoRC. PLoS Genet. 2014; 10:e1004157. 24651573 10.1371/journal.pgen.1004157 PMC3961174

[28] Smith A.K. , Wilkerson J.W., Knotts T.A. Parameterization of unnatural amino acids with azido and alkynyl R-Groups for use in molecular simulations. J. Phys. Chem. A. 2020; 124:6246–6253. 32614187 10.1021/acs.jpca.0c04605

[29] Abraham M.J. , Murtola T., Schulz R., Páll S., Smith J.C., Hess B., Lindahl E. GROMACS: high performance molecular simulations through multi-level parallelism from laptops to supercomputers. SoftwareX. 2015; 1–2:19–25.

[30] Van Der Spoel D. , Lindahl E., Hess B., Groenhof G., Mark A.E., Berendsen H.J.C. GROMACS: fast, flexible, and free. J. Comput. Chem. 2005; 26:1701–1718. 16211538 10.1002/jcc.20291

[31] Hart K. , Foloppe N., Baker C.M., Denning E.J., Nilsson L., MacKerell A.D. Optimization of the CHARMM additive force field for DNA: improved treatment of the BI/BII conformational equilibrium. J. Chem. Theory Comput. 2012; 8:348–362. 22368531 10.1021/ct200723y PMC3285246

[32] Kumar R. , Grubmüller H. do_x3dna: a tool to analyze structural fluctuations of dsDNA or dsRNA from molecular dynamics simulations. Bioinformatics. 2015; 31:2583–2585. 25838463 10.1093/bioinformatics/btv190

[33] Lu X. , Olson W.K. 3DNA: a software package for the analysis, rebuilding and visualization of three-dimensional nucleic acid structures. Nucleic Acids Res. 2003; 31:5108–5121. 12930962 10.1093/nar/gkg680 PMC212791

[34] Plotly Technologies Inc 2015; Collaborative data science Publisher: Plotly Technologies Inc. *Montr**é**al, QC*.

[35] Tsukiyama T. , Becker P.B., Wu C. ATP-dependent nucleosome disruption at a heat-shock promoter mediated by binding of GAGA transcription factor. Nature. 1994; 367:525–532. 8107823 10.1038/367525a0

[36] Pennings S. , Meersseman G., Bradbury E.M. Mobility of positioned nucleosomes on 5 s rDNA. J. Mol. Biol. 1991; 220:101–110. 2067009 10.1016/0022-2836(91)90384-i

[37] Imre L. , Simándi Z., Horváth A., Fenyőfalvi G., Nánási P., Niaki E.F., Hegedüs É., Bacsó Z., Weyemi U., Mauser R. et al. Nucleosome stability measured in situ by automated quantitative imaging. Sci. Rep. 2017; 7:12734. 28986581 10.1038/s41598-017-12608-9 PMC5630628

[38] Zuccheri G. , Scipioni A., Cavaliere V., Gargiulo G., De Santis P., Samorì B. Mapping the intrinsic curvature and flexibility along the DNA chain. Proc. Natl. Acad. Sci. U.S.A. 2001; 98:3074–3079. 11248034 10.1073/pnas.051631198 PMC30609

[39] Tolstorukov M.Y. , Colasanti A.V, McCandlish D.M., Olson W.K., Zhurkin V.B. A novel roll-and-slide mechanism of DNA folding in chromatin: implications for nucleosome positioning. J. Mol. Biol. 2007; 371:725–738. 17585938 10.1016/j.jmb.2007.05.048 PMC2000845

[40] Anselmi C. , Bocchinfuso G., De Santis P., Savino M., Scipioni A. A theoretical model for the prediction of sequence-dependent nucleosome thermodynamic stability. Biophys. J. 2000; 79:601–613. 10919995 10.1016/S0006-3495(00)76319-3 PMC1300961

[41] Widom J. Role of DNA sequence in nucleosome stability and dynamics. Q. Rev. Biophys. 2001; 34:269–324. 11838235 10.1017/s0033583501003699

[42] Ngo T.T.M. , Zhang Q., Zhou R., Yodh J.G., Ha T. Asymmetric unwrapping of nucleosomes under tension directed by DNA local flexibility. Cell. 2015; 160:1135–1144. 25768909 10.1016/j.cell.2015.02.001 PMC4409768

[43] Luo G.-Z. , Hao Z., Luo L., Shen M., Sparvoli D., Zheng Y., Zhang Z., Weng X., Chen K., Cui Q. et al. N6-methyldeoxyadenosine directs nucleosome positioning in tetrahymena DNA. Genome Biol. 2018; 19:200. 30454035 10.1186/s13059-018-1573-3 PMC6245762

[44] Zaichuk T. , Marko J.F. Single-molecule micromanipulation studies of methylated dna. Biophys. J. 2021; 120:2148–2155. 33838135 10.1016/j.bpj.2021.03.039 PMC8390797

[45] Pérez A. , Castellazzi C.L., Battistini F., Collinet K., Flores O., Deniz O., Ruiz M.L., Torrents D., Eritja R., Soler-López M. et al. Impact of methylation on the physical properties of DNA. Biophys. J. 2012; 102:2140–2148. 22824278 10.1016/j.bpj.2012.03.056 PMC3341543

[46] Osakabe A. , Adachi F., Arimura Y., Maehara K., Ohkawa Y., Kurumizaka H. Influence of DNA methylation on positioning and DNA flexibility of nucleosomes with pericentric satellite DNA. Open Biol. 2015; 5:150128. 26446621 10.1098/rsob.150128 PMC4632512

[47] Jimenez-Useche I. , Yuan C. The effect of DNA CpG methylation on the dynamic conformation of a nucleosome. Biophys. J. 2012; 103:2502–2512. 23260052 10.1016/j.bpj.2012.11.012 PMC3525854

