# Supplementary material for: Enhanced nucleosome assembly at CpG sites containing an extended 5-methylcytosine analogue
Source: Nucleic Acids Res. 2022 Jun 1;50(11):6549–61. doi: 10.1093/nar/gkac444 (PMC9226530; doi:10.1093/nar/gkac444)
Supplement: gkac444_Supplemental_Files [file gkac444_supplemental_files.zip › Movie 1_Tomkuviene et al 2022.docx]

Movie 1. An example of MD simulation of a 25-bp DNA with 4 ahyC bases. The snapshots were taken  from 10 ns to 100 ns of the simulation every 1 ns.
