# Supplementary material for: Enhanced nucleosome assembly at CpG sites containing an extended 5-methylcytosine analogue
Source: Nucleic Acids Res. 2022 Jun 1;50(11):6549–61. doi: 10.1093/nar/gkac444 (PMC9226530; doi:10.1093/nar/gkac444)
Supplement: gkac444_Supplemental_Files [file gkac444_supplemental_files.zip › Supplementary data_Tomkuviene et al 2022 final.pdf]

Supplementary Information

Designer Nucleosomes: enhanced nucleosome assembly at CpG sites containing an extended 5-methylcytosine analogue

Miglė Tomkuvienė[1], Markus Meier[2], Diana Ikasalaitė[1], Julia Wildenauer[2], Visvaldas Kairys[1], Saulius Klimašauskas[1,*] and Laura Manelytė[2,*]

[1] Institute of Biotechnology, Life Sciences Center, Vilnius University, Vilnius LT-10257, Lithuania

[2] Biochemistry III, University of Regensburg, Regensburg, Bavaria, DE-93053, Germany

* To whom correspondence should be addressed. Tel: +49 941 943 2867; Fax: +49 941 943 2474; Email: Laura.Manelyte@vkl.uni-regensburg.de. Correspondence may also be addressed to Saulius Klimašauskas. Email: saulius.klimasauskas@bti.vu.lt

Contents

Supplementary Materials and Methods

DNA oligonucleotides and substrates……………………………………………………………………… page 2-3

Fluorescence measurement of Ethidium binding to DNA………………………………………… page 3

Molecular dynamics simulations………………………………………………………………………………. page 3-4

Supplementary Figures and Tables

Modified substrates (Figure S1)………………………………………………………………………………. page 5

Purified proteins (Figure S2) ………………………………………………………………………………….. page 6

Restriction analysis of the nucleosomes on a single-modified 80-Widom DNA (Figure S3) page 7

Nucleosome assembly (Figure S4-S5)……………………………………………………………………… page 8-11

Nucleosome thermal and EtBr stability (Figure S6-S9)……………………………………………. page 12-15

Molecular dynamics simulation

Figure S10……………………………………………………………………………………………………… page 16

Figure S11……………………………………………………………………………………………………… page 17

Figure S12……………………………………………………………………………………………………… page 18-19

Figure S13……………………………………………………………………………………………………… page 19

Table S2 and S3…………………………………………………………………………………………… page 20

Figure S14……………………………………………………………………………………………………… page 21

Figure S15……………………………………………………………………………………………………… page 22

Figure S16……………………………………………………………………………………………………… page 23

Supplementary references……………………………………………………………………………………. page 23

Supplementary Materials and Methods

Templates for DNA substrate production: pUC18-601 (used to generate 80-Widom, 36-Widom and Widom substrates) and pUC19-Hsp70 (used to produce Hsp70 substrate) (kindly provided by G. Längst)

All oligonucleotides were purchased from Sigma-Aldrich.

Table S1. Oligonucleotides used.

**Name**	**Forward primer**	**Reverse primer**	**Length, bp**
80-Widom	5`-Cy3-CGATTAAGTTGGGTAACGC-3’<br>or 5’-Cy5-CGATTAAGTTGGGTAACGC-3`	5’-CCCGGGATGTATATATCTG-3`	229
Widom	5`-Cy3-CCTGGAGAATCCCGGTGC-3` or<br>5’-Cy5-CCTGGAGAATCCCGGTGC -3`	5’-CGGGATGTATATATCTGACACG-3’	147
36-Widom	5`-Cy3-CGACGTTGTAAAACGACGGC-3` or<br>5’- CGACGTTGTAAAACGACGGC-3`	5`-TAATCCCCTTGGCGGTTAAAAC-3`	151
Hsp70	5`-Cy3-AATTCAGATCTGAATTGACGTTCCGT-3`<br>or 5`-Cy5-AATTCAGATCTGAATTGACGTTCCGT-3`	5’-CGGATCCCACGATAAGCATAAC-3’	361
40-Widom-40	5`-Cy5-GTTGTAAAACGACGGCCAGTGAAT-3`	5`-GCCAAGCTTGCATGCCTG-3`	227

DNA substrates

The M.SssI targets CG are highlighted in yellow, the M.HhaI target G**C**GC is underlined (the first C in the target sequence is modified). The CG sites at the ends of the substrate are believed to escape modification.

80-Widom DNA

CGATTAAGTTGGGTAACGCCAGGGTTTTCCCAGTCACGACGTTGTAAAACGACGGCCAGTGAATTCG
AGCTCGGTACCCGGGTGGAGAATCCCGGTGCCGAGACCGCTCAATTGGTCGTAGCAAGCTCTAGCA
CCGCTTAAACGCACGTACGCGCTGTCCCCCGCGTTTTAACCGCCAAGGGGATTACTCCCTAGTCTC
CAGGCACGTGTCAGATATATACATCCCGGG

36-Widom

CGACGTTGTAAAACGACGGCCAGTGAATTCGAGCTCGGTACCCGGGTGGAGAATCCCGGTGCCGA
GACCGCTCAATTGGTCGTAGCAAGCTCTAGCACCGCTTAAACGCACGTACGCGCTGTCCCCCGCGT
TTTAACCGCCAAGGGGATTA

Widom

GGTGGAGAATCCCGGTGCCGAGACCGCTCAATTGGTCGTAGCAAGCTCTAGCACCGCTTAAACGCA
CGTACGCGCTGTCCCCCGCGTTTTAACCGCCAAGGGGATTACTCCCTAGTCTCCAGGCACGTGTCA
GATATATACATCCCG

Hsp70

AATTCAGATCTGAATTGACGTTCCGTCGACGAAGCGCCTCTATTTATACTCCGGCGCTTCTTTTCGCG
AACATTCGAGGCGCGCTCTCTCGAAGCAACGAGAACAGTGTGCCGTTTACTGTGCGACAGAGTGAG
AGAGCAATAGTACAGAGAGGGAGAGTCACAAAACGAATAGAGAATAACGGCCAGAGAAATTTCTCGA
GTTTTCTTTCTGCCAAACAAATGACCTACCGCAACAACCAGTTTGTTTTGGGATTCTAGAATATTCGCT
TTATTTTGGAAATTTCTTTATAAATACGGCTGCTTAAATTAATTATGGTAGAGATAATCGCAGAGCTTG
GTTATGCTTATCGTGGGATCCG

40-Widom-40 (was used only unmodified)

GTTGTAAAACGACGGCCAGTGAATTCGAGCTCGGTACCCGGGTGGAGAATCCCGGTGCCGAGACC
GCTCAATTGGTCGTAGCAAGCTCTAGCACCGCTTAAACGCACGTACGCGCTGTCCCCCGCGTTTTAA
CCGCCAAGGGGATTACTCCCTAGTCTCCAGGCACGTGTCAGATATATACATCCCGGGGATCCTCTAG
AGTCGACCTGCAGGCATGCAAGCTTGGC

Fluorescence measurement of Ethidium binding to DNA

To monitor DNA-EtBr interaction, the increase of EtBr fluorescence upon DNA binding was measured (1). Briefly, Widom and 36-Widom substrates were ahyC-modified using eM.SssI. Modified and unmodified substrates were then diluted 2x stepwise in the nucleosome assembly buffer containing 0,3 µM EtBr, in a black 96-well plate. 100 µl dilutions were measured at Ex 300 nm and Em 605 nm wavelengths in a BioTek Synergy 4 Microplate Reader. The assay was repeated in triplicate.

Molecular dynamics simulations

The CHARMM-compatible topology for the modified ahyC nucleotide tail was built by hand based on azido and alkynyl group parameters optimized by Smith *et al.* (2). Ethidium ion parameters were generated using CGenNFF v. 4.4 (3). Because of the lack of bromide parameters they were replaced by chloride ions for the DNA/ethidium simulation. MD simulation box with the nucleic acid in the center was set to dodecahedral, with 10 Å distance between the surface of the box and the nearest nucleic acid atom before the start of the simulation. Besides the inserted nucleic acid, the box was filled with TIP3P water molecules (4), and also with Na^+ and Cl^- ions to neutralize the system and to create an overall 0.15 M concentration of salt. For the 5 different simulation replicas, different random seeds were used during the insertion of ions step, leading to slightly different environments of the nucleic acid for each replica. For the simulations that explored ethidium ion interactions with DNA, the setup was very similar as described

above and previously (5). 4 ethidium ions were added to the initial simulation box. Initially all ethidium molecules were positioned near the middle part of DNA duplex at a 25 Å distance from the axis of DNA at approximately equal distances from each other, which translated to about 5 Å initial distance from DNA to ethidiums. In the DNA/ethidium runs the DNA was either freely relaxed, or bent to reproduce the DNA curvature observed in nucleosomes. 5 replicas were run fore each of these cases. The bending was imposed onto DNA by applying a harmonic constraint with PLUMED v. 2.7.0 plugin (6): the distance from the axis, connecting pseudoatoms located midway between the C1‘ atoms of both terminal base pairs, to the analogous pseudoatom in the middle base pair of DNA was biased to 17 Å with 200 kJ·mol^{-1}nm^{-1} force constant.

The assembled system in each case was first subjected to a steepest descents minimization until the maximum force on any of the atoms decreased to less than 1000 kJ·mol^{-1}nm^{-1}. Afterward, the system was subjected to a 0.1 ns *NVT* equilibration using 2 fs time steps at 293K temperature, with the position restraints imposed on nucleic acid atoms. The production runs were done using 2 fs time steps under *NPT* conditions at 293 K and 1 bar pressure. The temperature coupling at both *NVT* and *NPT* steps used a V-rescaling algorithm with used τ_T = 0.1 ps time constant, using nucleic acid and atoms and the rest of the system as two coupled groups. The pressure coupling for the *NPT* run was done using the Parrinello-Rahman algorithm, with the time constant τ_p = 2 ps, and 4.5x10^{-5} bar^{-1} isothermal compressibility for water. The run used the Verlet cutoff scheme. Particle Mesh Ewald (PME) algorithm was used for electrostatic interactions, with a 1.2 nm cutoff and using 4th order cubic interpolation with 0.16 nm grid spacing for Fast Fourier Transform. A single 1.2 nm cutoff was also used for van der Waals interactions. To reduce fraying of the base pairs at both ends of the nucleic acid duplex during the production run, 400 kJ·mol^{-1}nm^{-1} harmonic restraints were added between the terminal base pair nucleotide atoms connected with hydrogen bonds, with the restrained distances equal to 0.18 nm. The restraints were imposed via PLUMED v. 2.7.0 plugin (6). The nucleic acid conformations were recorded every 10 ps. The length of the production runs for the DNA duplex in the solution were 100 ns. To gather conformational statistics for these MD simulations, the last 90 ns of each 100 ns production run was used, resulting in 9001 conformations for each replica. The MD simulation trajectories were analyzed with the do_x3dna package (7) that uses the 3DNA program (v. 2.1) (8) to analyze individual MD snapshots. The length of the production runs involving DNA and ethidium ions was 1.2 µs for each replica of the simulation. Because ethidium intercalation events in our simulations occurred randomly and irreversibly, this enabled us to calculate intercalation halftimes $\tau_{1/2}$ by fitting intercalation onset times into the exponential decay curves using R statistical package (v. 4.1.2).

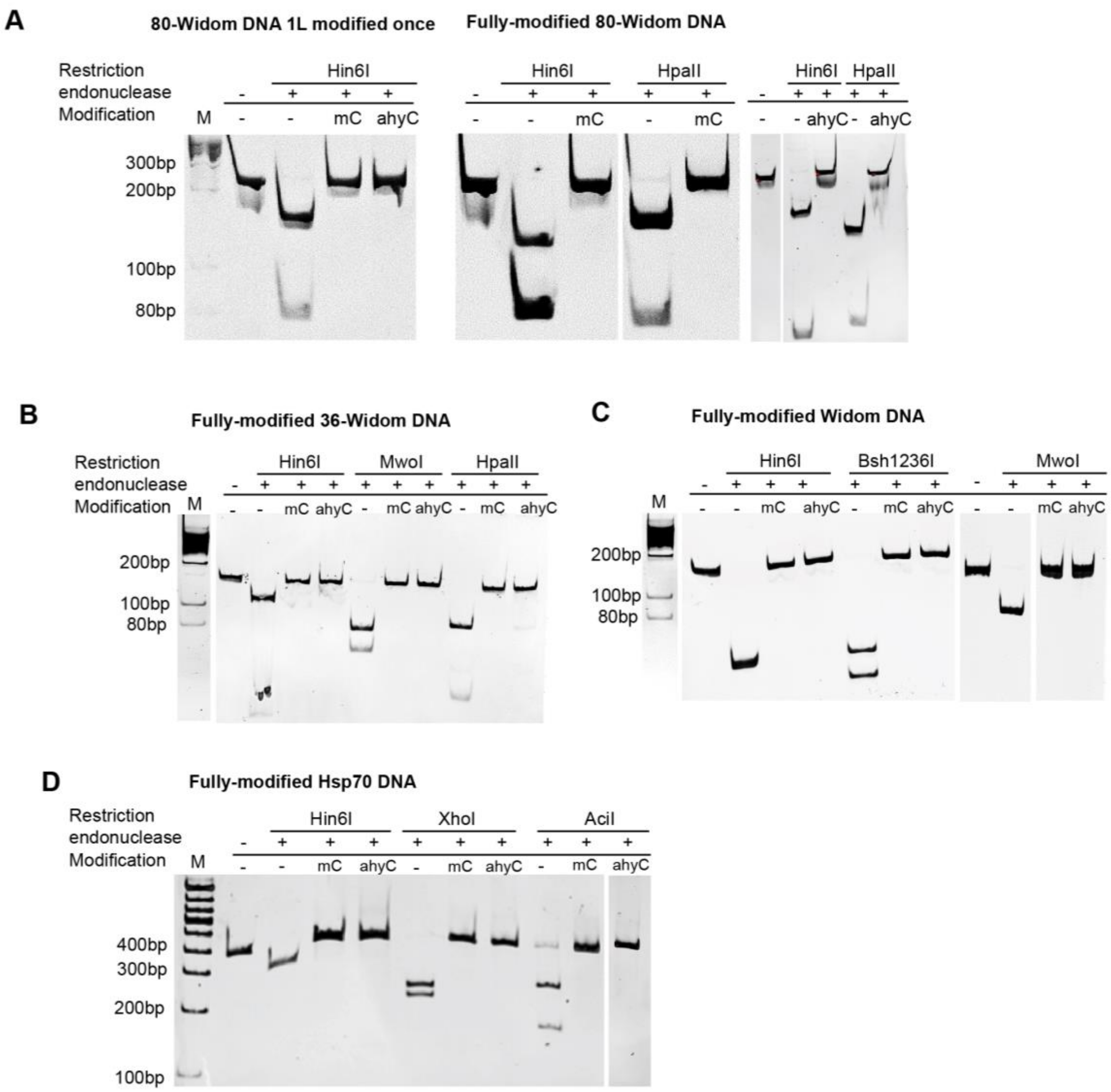

Figure S1. Restriction digestion analysis of the modified substrates. For the substrates modified at CG sites restriction endonucleases Bsh1236I (Widom fragment), Hin6I (for 36-Widom, Widom, 80-Widom and Hsp70), HpaII (for 36-Widom, 80-Widom and Hsp70) and MwoI (for 36-Widom, Widom and Hsp70) were used. For 80-Widom DNA modified at one site, R.Hin6I was used.

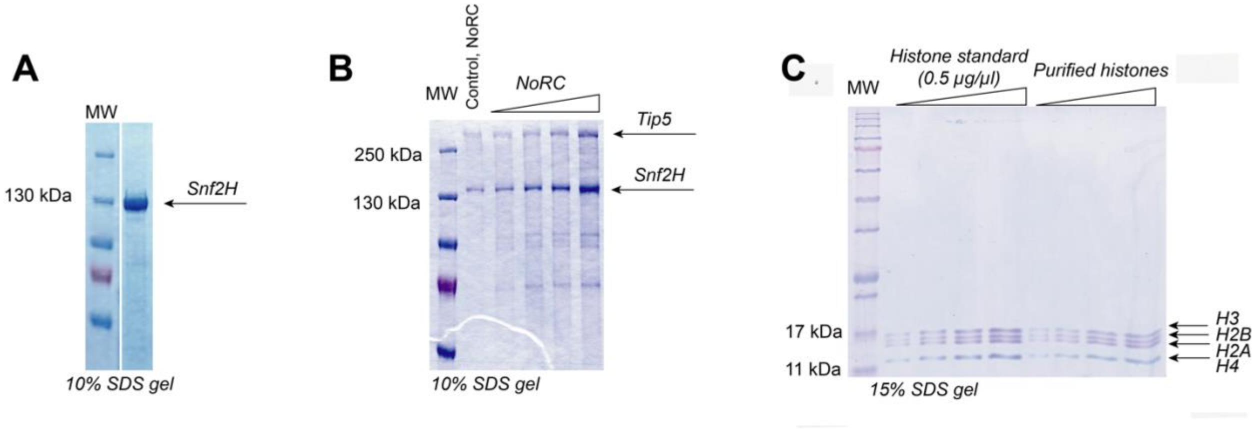

Figure S2. SDS-PAGE gels showing the purified proteins used in this study. (**A**) 750 ng of purified Snf2H and (**B**) different amounts of purified NoRC (123 ng/µl), which consist of Snf2H and Tip5, and a control NoRC were loaded onto 10% Tris-glycine SDS gels. MW lane in (A) and (B) is Thermo Scientific™ PageRuler™ Plus Prestained Protein Ladder. (**C**) Different amounts of histone standard (Sigma-Aldrich H9250) and our purified calf thymus histones (0.4 µg/µl) were loaded onto 15% Tris-glycine SDS gel. MW lane is BLUeye Prestained Protein Ladder (GeneDirex).

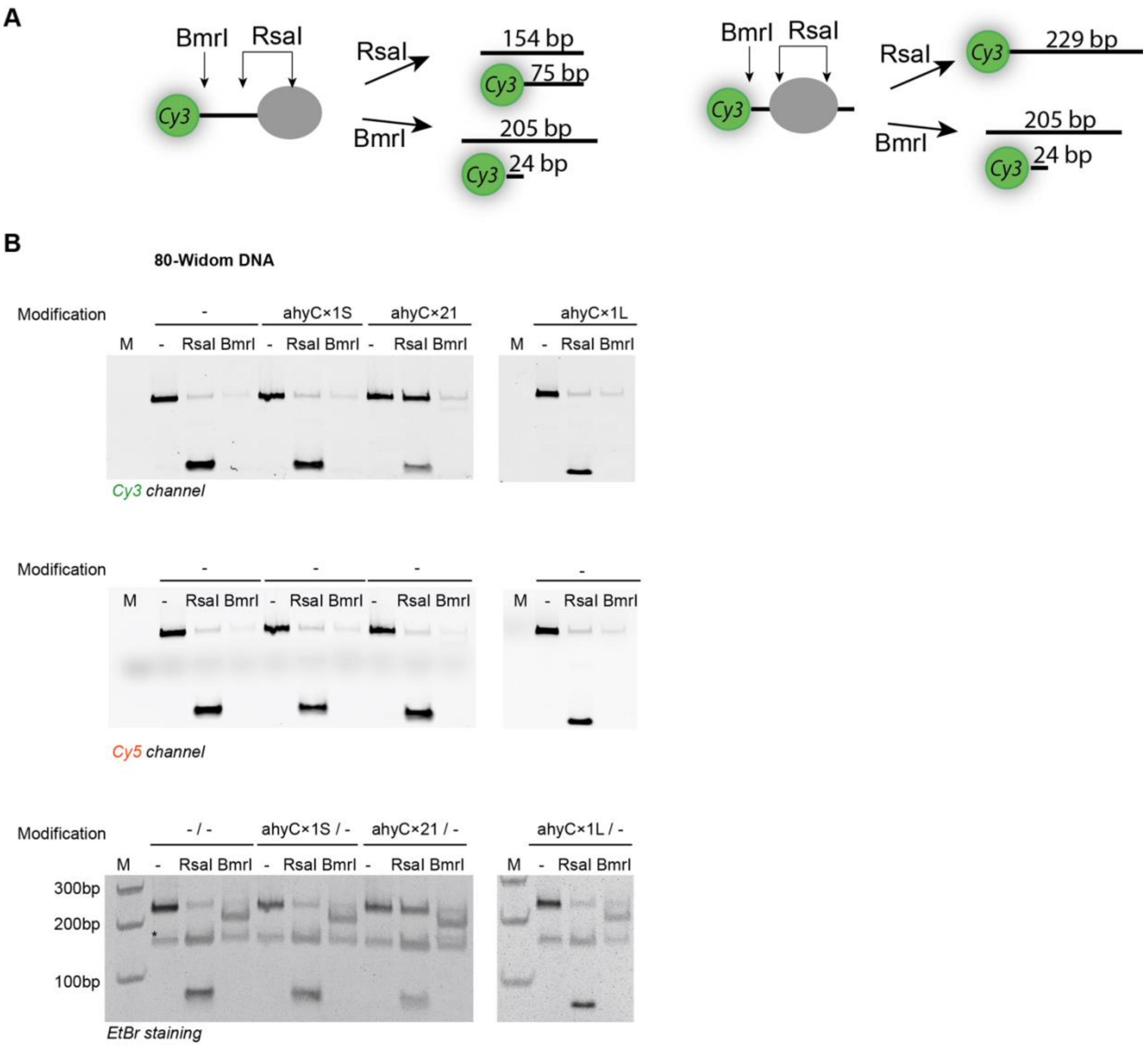

Figure S3. Restriction endonuclease probing of the nucleosome positioning on the 80-Widom DNA substrate modified at a single site. (**A**) Schematic representation of the restriction-digestion pattern of the nucleosomes assembled onto 80-Widom DNA. (**B**) The non-modified or ahyC containing nucleosomes (1S, 1L or 21) were digested with RsaI and BmrI restriction-endonucleases. The gel images of the fluorescence scan (two upper gel images) or ethidium bromide staining (lower gel image) are shown.

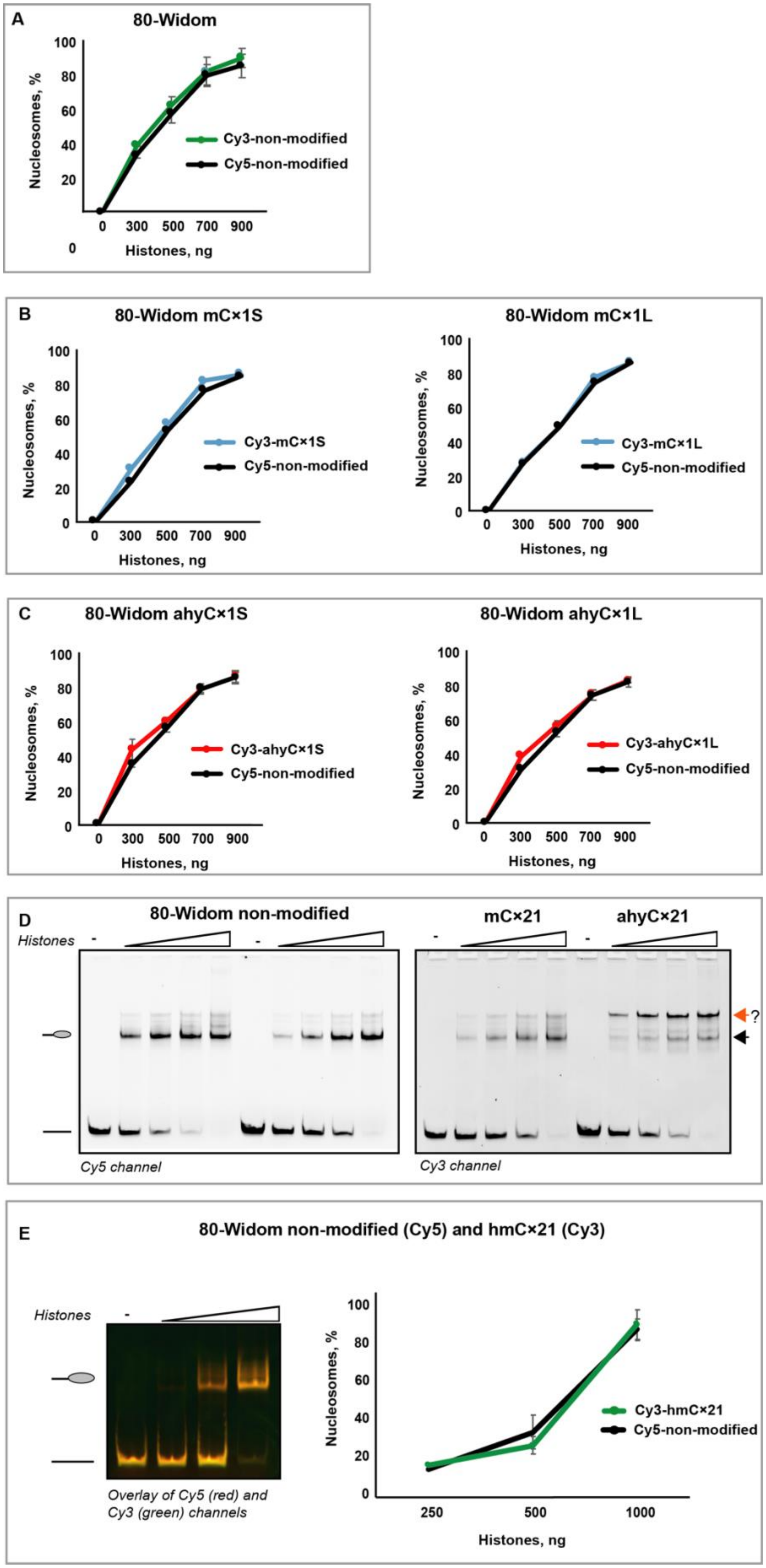
A
80-Widom
Nucleosomes, %
Histones, ng
Cy3-non-modified
Cy5-non-modified
B
80-Widom mC×1S
80-Widom mC×1L
Cy3-mC×1S
Cy5-non-modified
Cy3-mC×1L
Cy5-non-modified
C
80-Widom ahyC×1S
80-Widom ahyC×1L
Cy3-ahyC×1S
Cy5-non-modified
Cy3-ahyC×1L
Cy5-non-modified
D
80-Widom non-modified
mC×21
ahyC×21
Histones
Cy5 channel
Cy3 channel
E
80-Widom non-modified (Cy5) and hmC×21 (Cy3)
Histones
Overlay of Cy5 (red) and Cy3 (green) channels
Cy3-hmC×21
Cy5-non-modified

Figure S4. **Nucleosome assembly efficiency upon increasing concentrations of the histones.** (**A**) Cy3- and Cy5-labelled 80-Widom DNA assembled into nucleosomes; (**B**) Nucleosome assembly efficiency of Cy3-labelled 80-Widom DNA that was methylated once in the Widom DNA (mC×1S, right graph) or once in the linker DNA (mC×1L, left graph) and mixed with Cy5-labelled non-modified DNA substrate; (**C**) Nucleosome assembly efficiency of Cy3-labelled 80-Widom DNA that was azidohexynylated once in the Widom DNA (ahyC×1S, right graph) or once in the linker DNA (ahyC×1L, left graph) and mixed with Cy5-labelled non-modified DNA substrate. Data points are the average of at least three independent experiments, with standard deviation; (**D**) Gel images of one pot nucleosome assembly on 80-Widom non-modified and with mC×21 or ahyC×21. Black arrow indicates the end-positioned nucleosome species, whereas red arrow indicates preferentially formed new species on the ahyC×21 modified 80-Widom DNA substrate; (**E**) Gel image and graphical representation of one pot nucleosome assembly on 80-Widom non-modified and fully hydroxymethylated (hmC×21) DNA. Data points are the average of three independent experiments, with standard deviation.

A

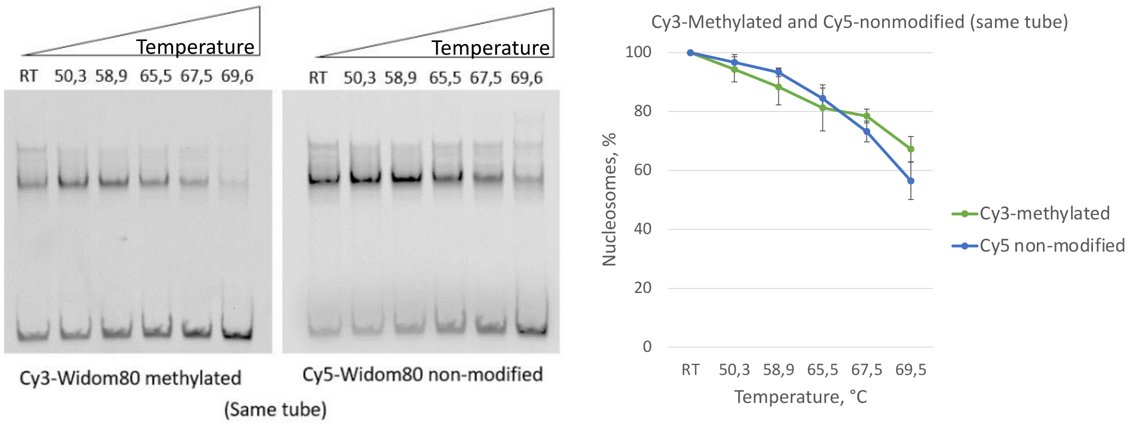

B

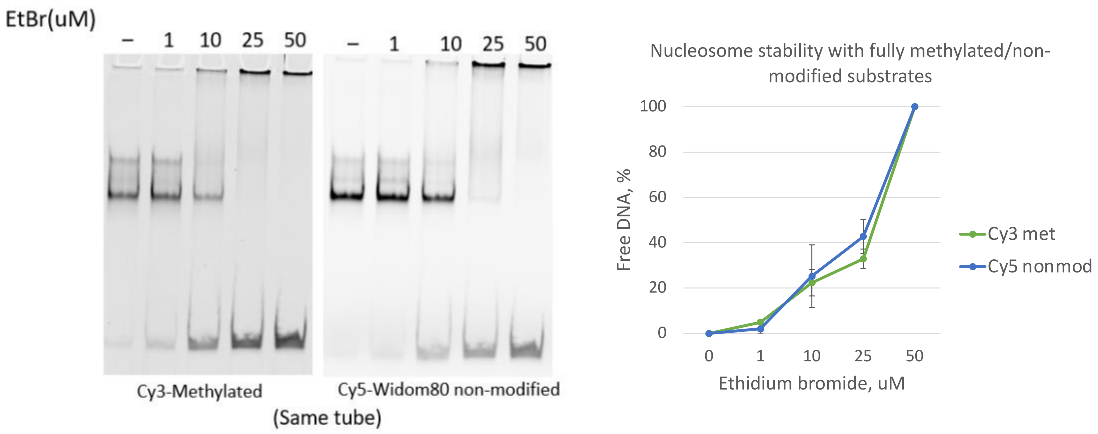

(continued on the next page)

(continued)

C

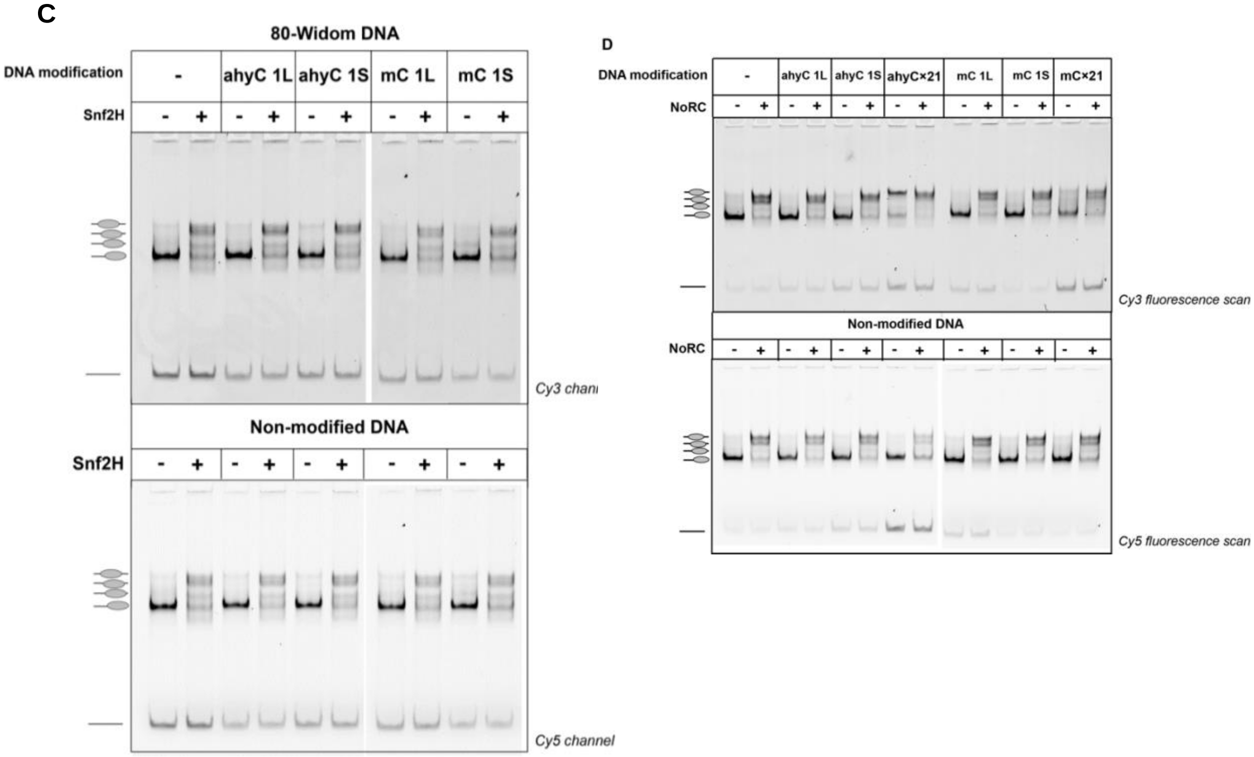

Figure S5. Methylation or single ahyC DNA modification does not affect the nucleosome stability and repositioning by chromatin remodeler Snf2H. Thermal (**A**) and Ethidium bromide (**B**) stability of the methylated and non-modified nucleosomes assembled on the 80-Widom DNA template. Data points are the average of at least three independent experiments, with standard deviation. Chromatin remodeler Snf2H (**C**) or NoRC (**D**) repositions nucleosomes to similar positions on non-modified and once modified nucleosome substrates. Either ahyC or mC modification was placed at one site (linker, L or Widom sequence, S).

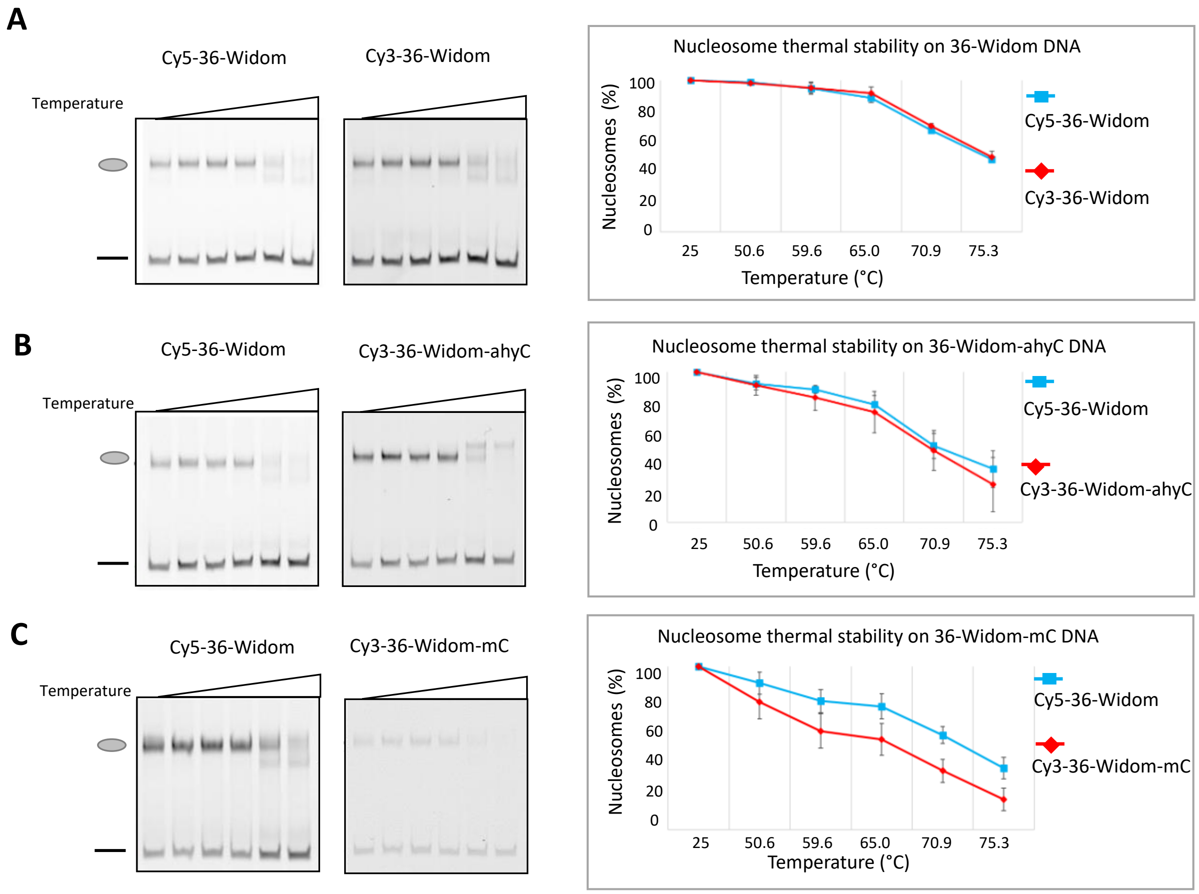

Figure S6. Thermal shift assays of 36-Widom-nucleosomes. Thermal shift assay with (**A**) Cy5- and Cy3-labelled nucleosomes assembled 36-Widom DNA showing that the nucleosomes containing both fluorophores behave similarly; (**B**) Cy5-36-Widom and Cy3-36-Widom-ahyC; (**C**) Cy5-36-Widom and Cy3-36-Widom-mC. The gel images of the fluorescence scans and graphical representations are shown. Data points are the average of at least three independent experiments, with standard deviation.

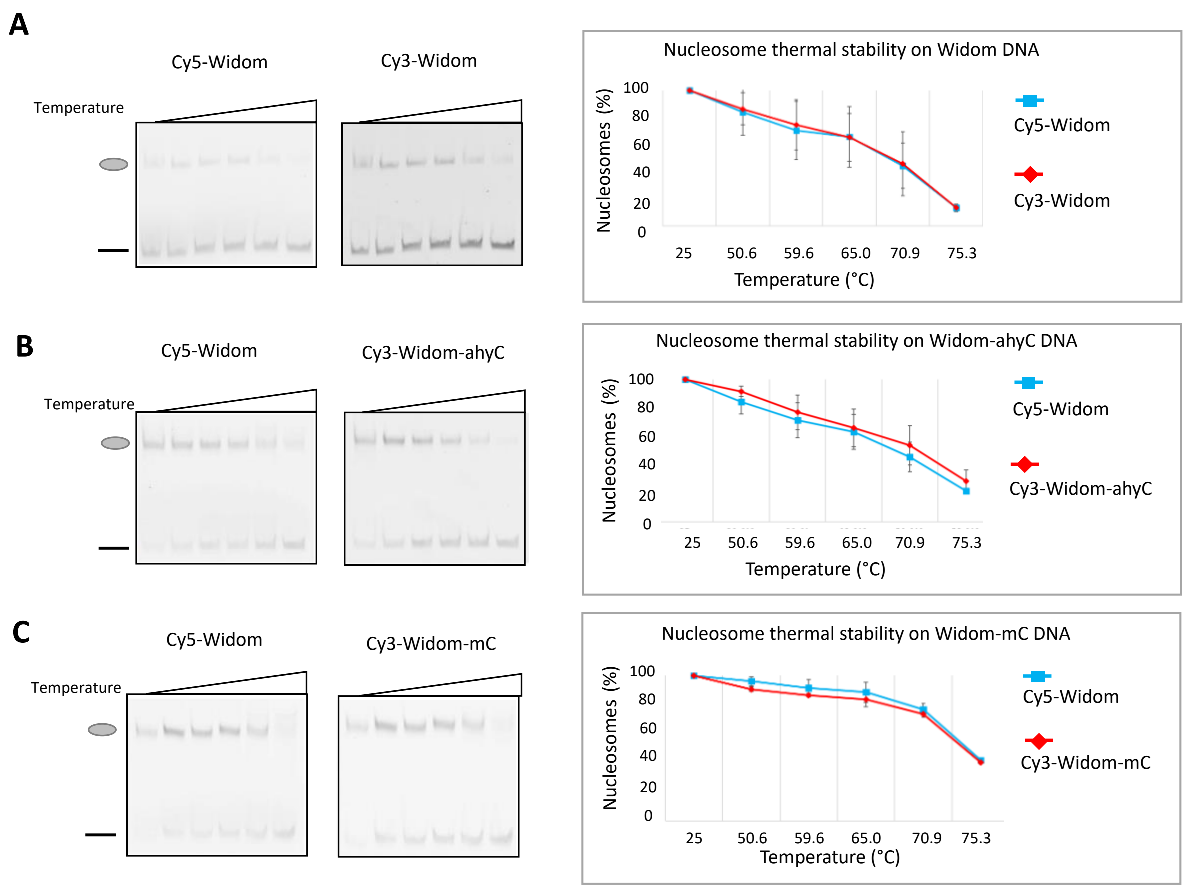

Figure S7. Thermal shift assays of Widom-nucleosomes. Thermal shift assay with (**A**) Cy5- and Cy3-labelled nucleosomes assembled Widom DNA showing that the nucleosomes containing both fluorophores behave similarly; (**B**) Cy5-Widom and Cy3-Widom-ahyC; (**C**) Cy5-Widom and Cy3-Widom-mC. The gel images of the fluorescence scans and graphical representations are shown. Data points are the average of at least three independent experiments, with standard deviation.

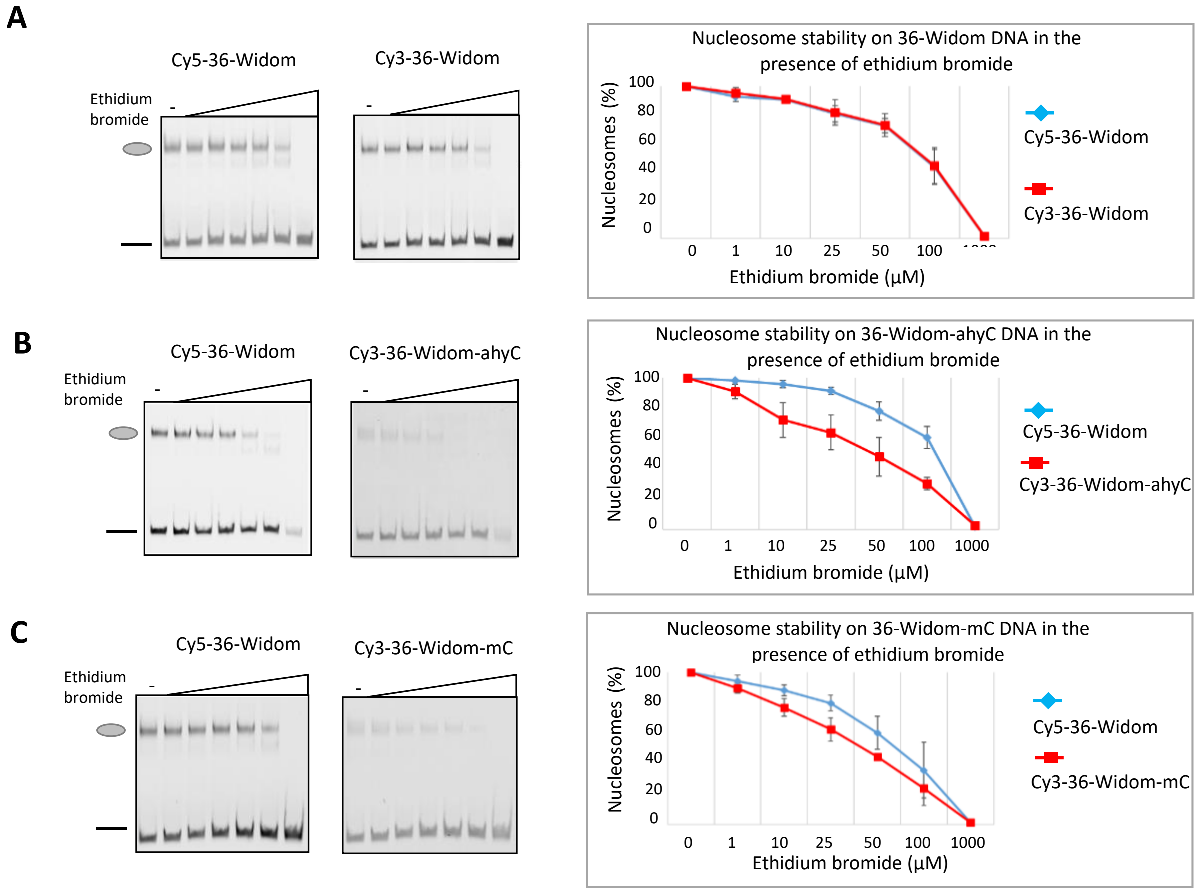

Figure S8. EMSA analysis of EtBr interaction with 36-Widom-nucleosomes. (**A**) EtBr assay with Cy5-36-Widom nucleosomes serving as reference for EtBr assay with wt Cy3-36-WIDOM nucleosomes. EtBr assay with wt Cy5-36-WDOM nucleosomes serving as reference for (**B**) EtBr assay with Cy3-36-WIDOM-ahC nucleosomes. (**C**) EtBr assay with wt Cy5-36-WIDOM nucleosomes serving as reference for EtBr assay with Cy3-36-WIDOM-mC nucleosomes. Data points are the average of three independent experiments, with standard deviation.

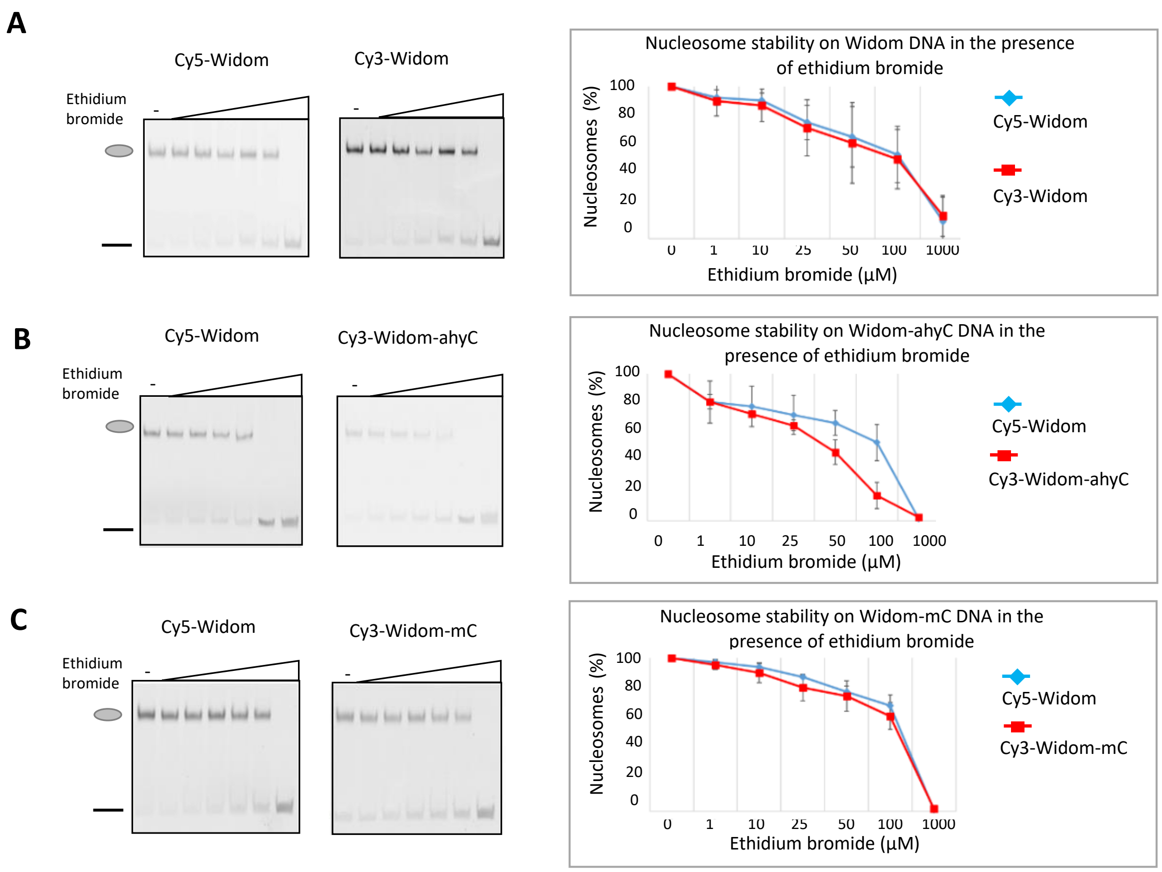

Figure S9. EMSA analysis of WIDOM nucleosomes in the presence of EtBr. (**A**) EtBr assay with Cy5-WIDOM-nucleosomes serving as reference for EtBr assay with Cy3-WIDOM nucleosomes. (**B**) EtBr assay with Cy5-WIDOM nucleosomes serving as reference for EtBr assay with Cy3-WIDOM-ahyC nucleosomes. (**C**) EtBr assay with Cy5-WIDOM nucleosomes serving as reference for EtBr assay with Cy3-WIDOM-mC nucleosomes. Data points are the average of three independent experiments, with standard deviation.

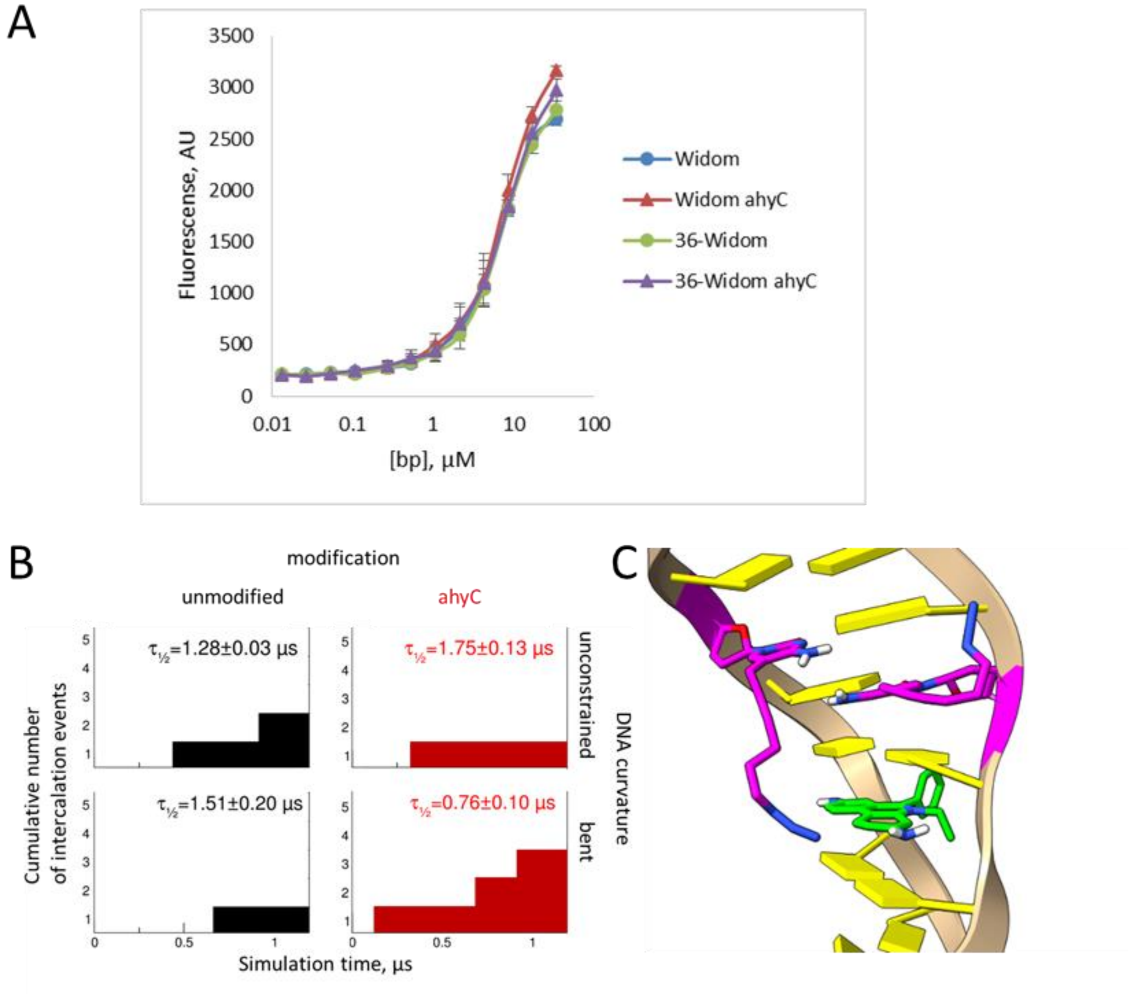

Figure S10. Interaction of ethidium bromide with ahyC DNA substrates. (A) Free DNA substrate (Widom or 36-Widom, unmodified or with multiple ahyC as shown) was gradually added to a buffer containing 0.3 µM EtBr, and fluorescence (excitation/emission at 300/605 nm) was monitored at each DNA concentration (shown in total bps). Plotted data points are mean values with standard deviation from three independent experiments. **(B)** MD simulation of EtBr intercalation in a 25-bp DNA in five parallel simulation runs for ahyC or unmodified DNA, each unrestrained (straight DNA, upper panel) or bent at a radius corresponding to that of DNA wrapped in a nucleosome (bent DNA, lower panel). The interaction was quantified by measuring the time points and number of cumulative intercalation events during 5 runs of 1.2 µs simulations (derived intercalation halftimes are shown for each DNA). **(C)** An illustrative example of intercalated ethidium ion in pre-bent ahyC DNA observed in a MD simulation. Ethidium and ahyC are colored green and magenta, respectively. Intercalation takes place between unmodified base pairs. The side chain of an ahyC residue is seen interacting via its azide group with the bound ethidium ion.

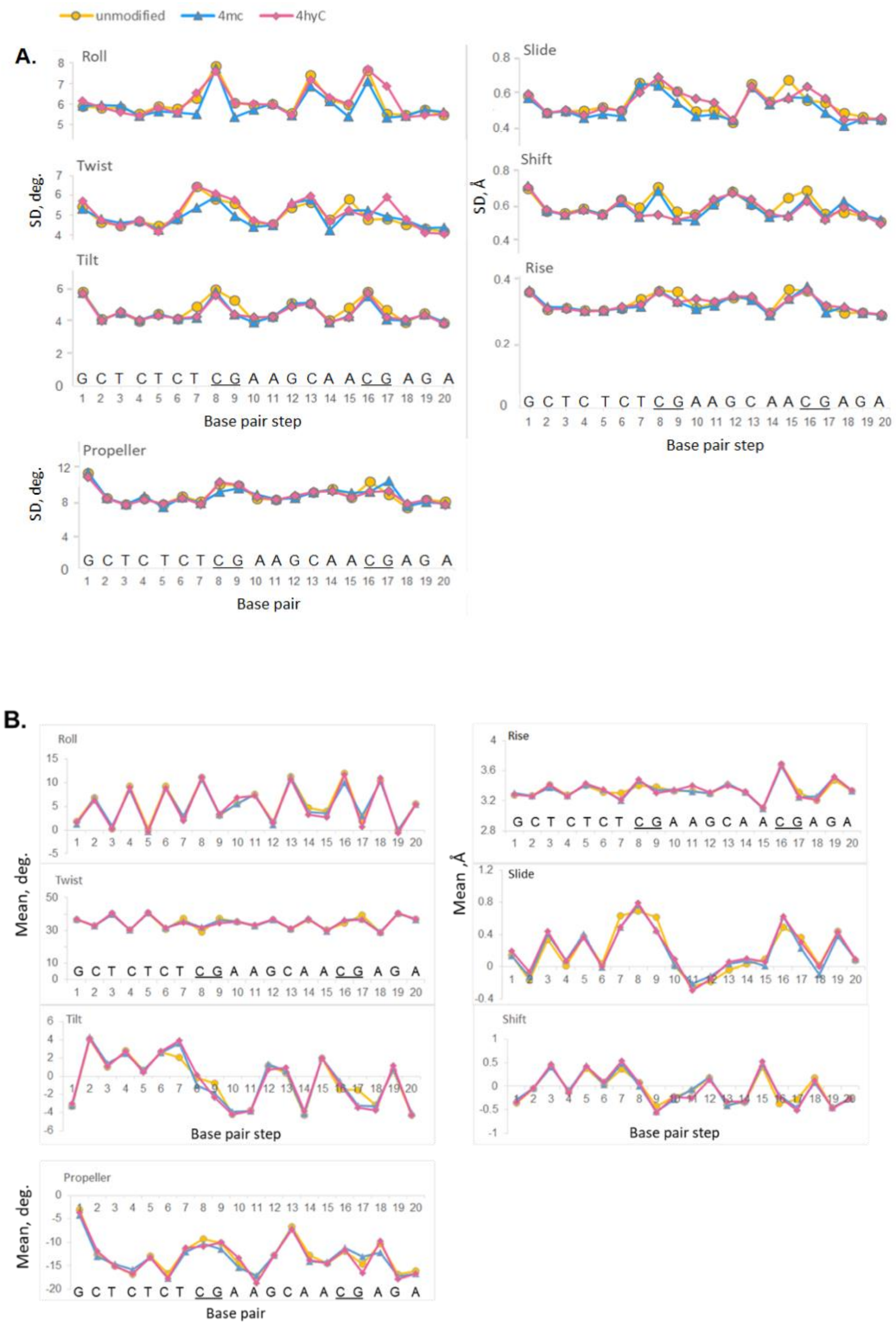

Figure S11. **Standard deviation (A) and mean (B) of DNA parameters in unmodified, methylated (4mC) and ahyC-modified (4ahyC) DNA**. 25-bp DNA fragment containing two CG sites (bp no. 8-9 and 16-17, underlined) was subjected to 100 ns molecular dynamics simulation followed by helical analysis with 3DNA software. The SD are shown for 20 internal base pairs, excluding the terminal four residues due to end fraying.

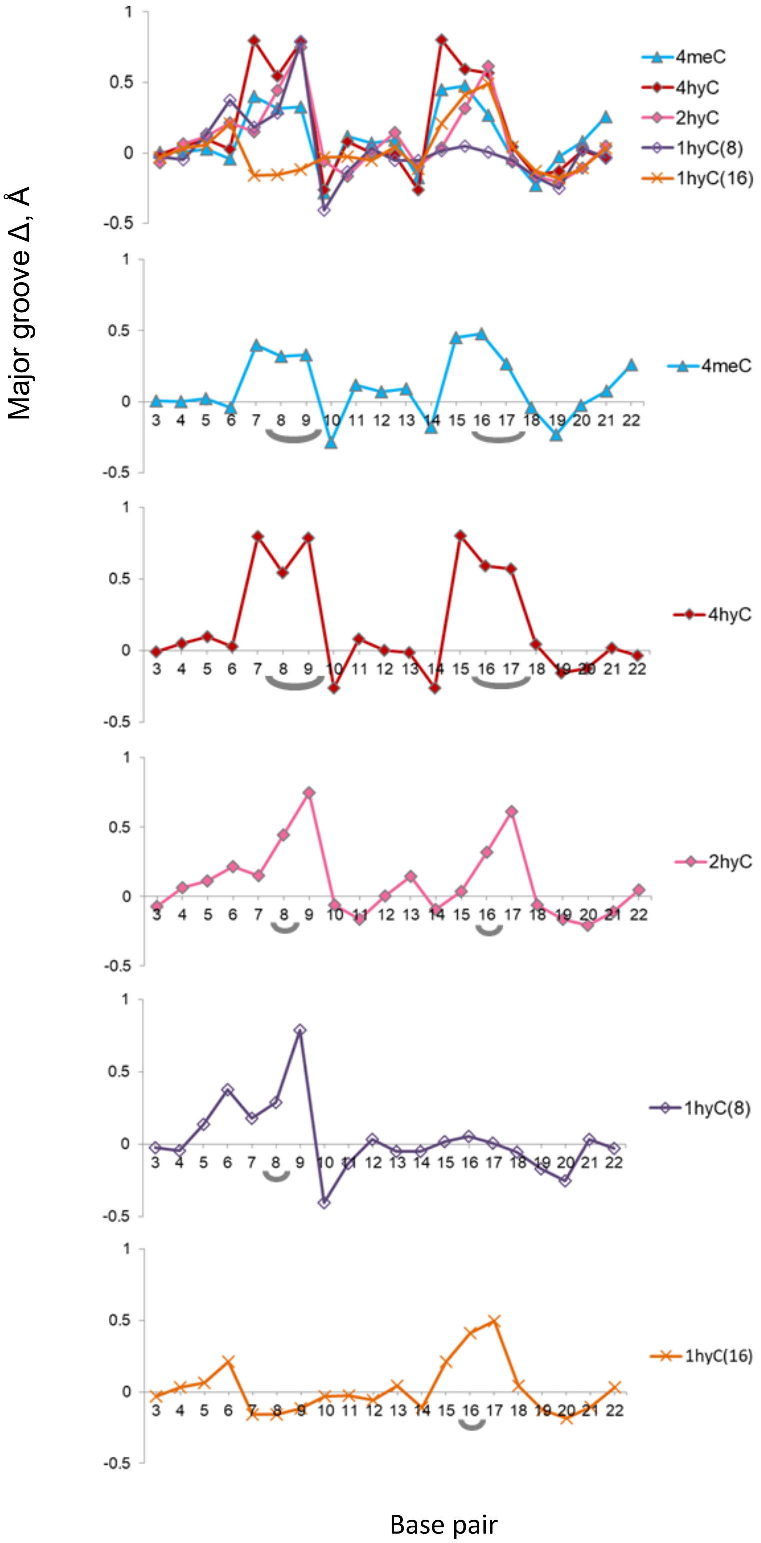

(continued on the next page)

(continued)

Figure S12. **Widening of the major groove in the modified DNA fragments**. 25-bp DNA fragment containing two CG sites (bp no. 8-9 and 16-17) was subjected to 100 ns molecular dynamics simulation. The CG sites were either fully or hemi-modified, the modified base pairs are marked with asterisks. The helical parameters were analyzed using 3DNA software. The delta of the major groove parameter (modified vs unmodified DNA) is shown along the DNA fragment length (the end residues removed due to extensive fraying).

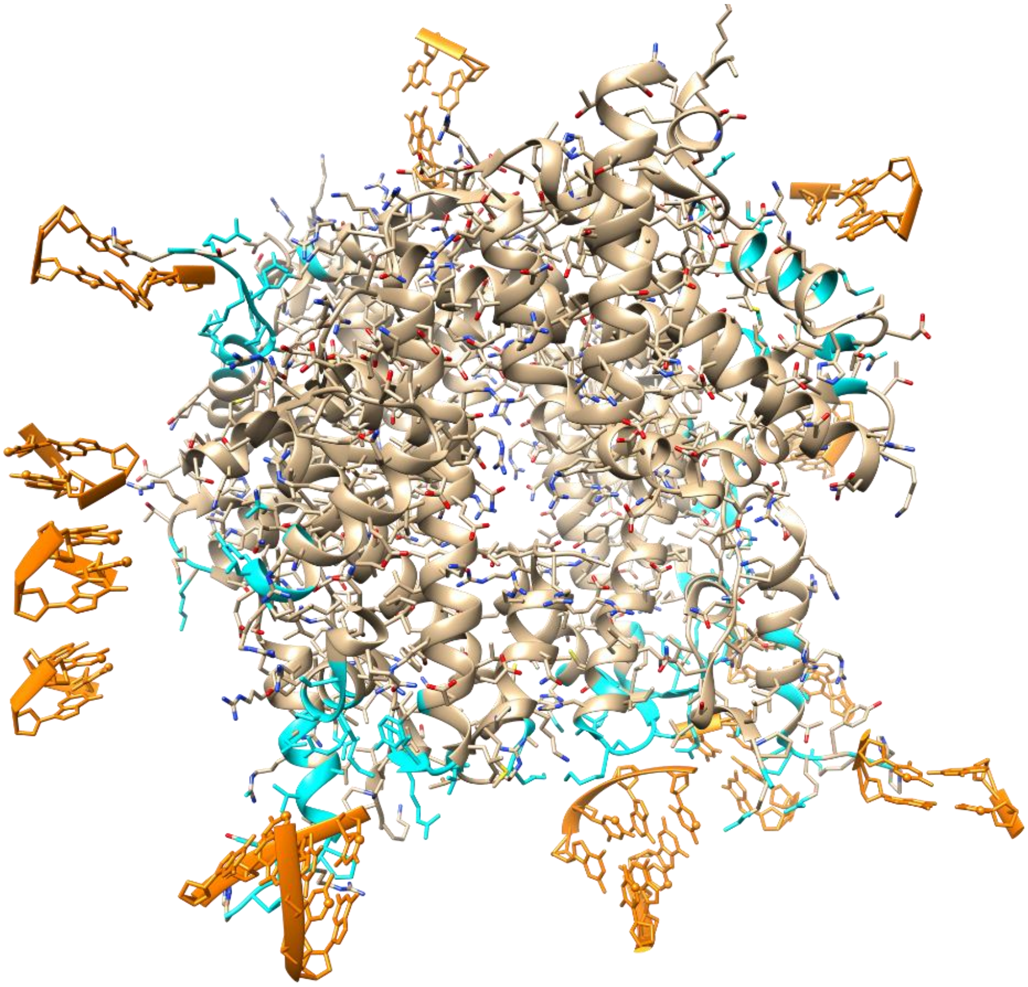

Figure S13. **Histone protein atoms (cyan) potentially reachable by azidohexynyl tails of ahyC DNA**. Potential contact zones were assigned to histone atoms located within 12 Å (approximate length of the extended azidohexynyxl side chain) from C5 atoms of ahyC (small orange balls). Only CpG sites of the nucleosome DNA are displayed for clarity (orange). PDB id: 3LZ0

Table S2

Interactions of ahyC azide in 1ahyC(8) substrate. Strand A – the one with ahyC residue, strand B – complementary. Interaction threshold 4.5 Å. The shown contacts are the sum of time points from all the five independent repeats of the molecular dynamics simulations (every trajectory has overall 9001 time points).

No.	strand	base	number of interactions
5	A	T	2
6	A	C	1313
7	A	T	3744
8	A	ahyC	3979 (own-base interaction)
9	A	G	1711
10	A	A	1895
11	B	G	47
12	B	C	585
11	B	T	5570
10	B	T	3273
9	B	C	2257
8	B	G	42
7	B	A	6

Table S3

Azide-DNA interaction events are very frequent in 4ahyC substrate. Number of time points in 4ahyC molecular dynamics trajectory with either no azide group-DNA interactions or with at least one group interacting. All the trajectories have an overall sum of 9001 time points.

MD simulation repeat	Any azide group touches DNA (distance less than 4.5 Å)	All azide groups are exposed to the solvent (distance more than 4.5 Å)	Percent of time points with at least one touching conformation
1	8646	355	96%
2	8862	139	98%
3	8628	373	96%
4	8739	262	97%
5	8427	574	94%

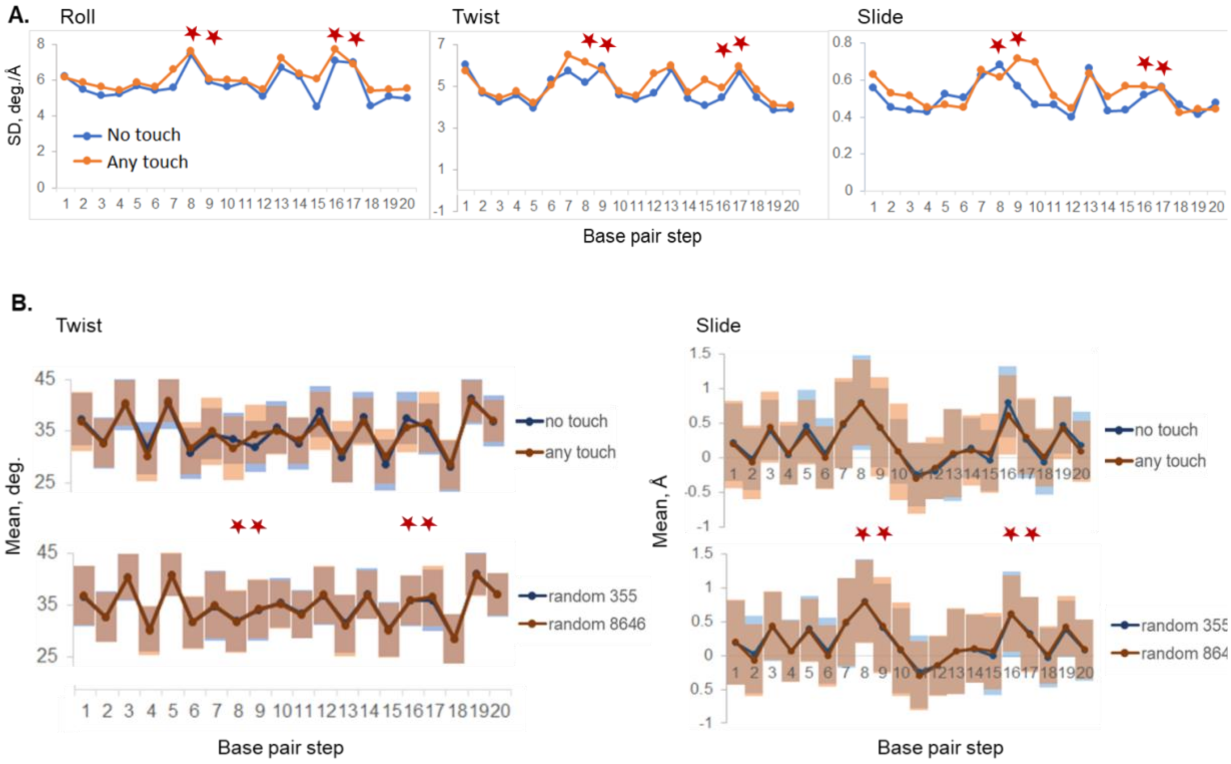

Figure S14. **Dynamic DNA structure deviations are promoted by temporary ahyC goup interactions with DNA.** 9001 time points of 4ahyC molecular dynamics simulation trajectory were sorted into two groups: the ones with all the four ahyC azide groups exposed to the solvent (more than 4.5 Å away from DNA) – “no touch” (355 points); and the ones that had at least one ahyC azide group close to DNA (less than 4.5 Å) – “any touch” (8646 points). (**A**) Standard deviation of Roll, Twist and Slide parameters in the groups of time points with either all the azide groups out in the solvent or touching any neighbour site in DNA. (**B**) Mean Twist and Slide values with standard deviation at every base pair – upper panel. The same starting set of time points was randomly divided into two groups of the same size – lower panel. The ahyC modifications are marked with asterisks.

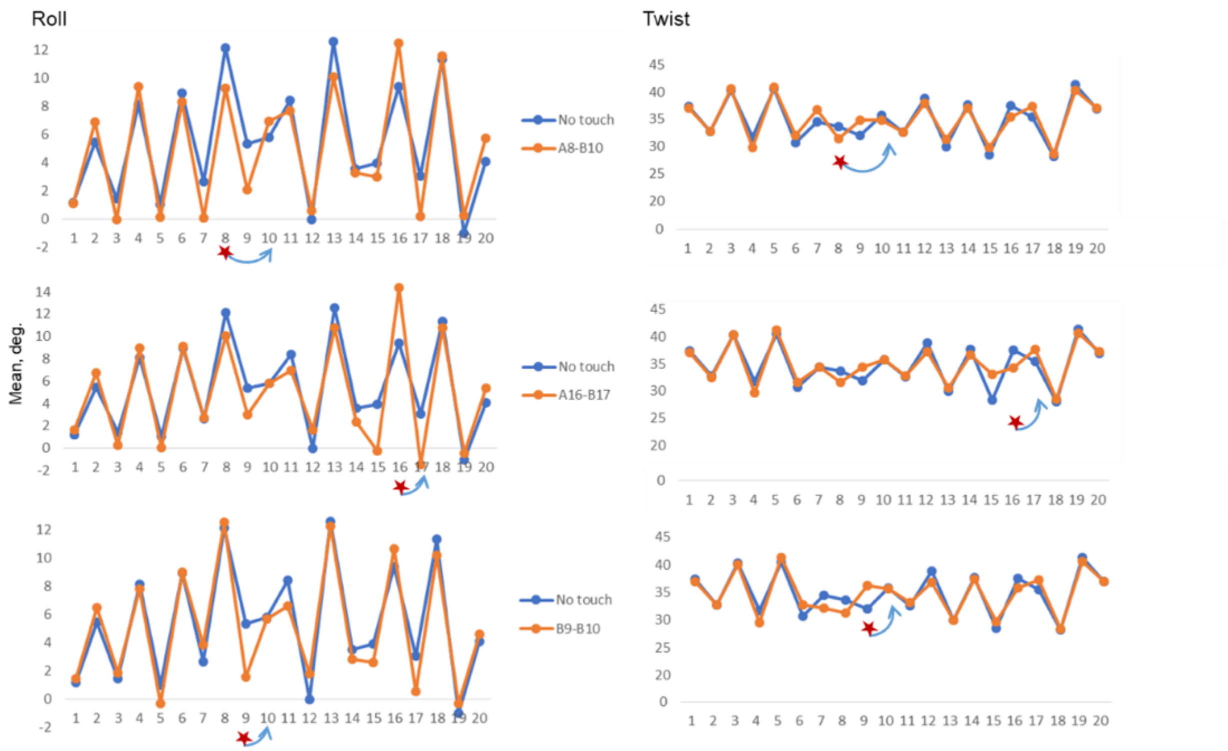

Figure S15. Structural effects of ahyC-DNA interactions observed at individual time points. 9001 time points of 4ahyC molecular dynamics simulation trajectory were sorted into two groups: the ones with all the four ahyC azide groups exposed to the solvent (more than 4.5 Å away from DNA) – "no touch"; and the ones that had a selected azide group interaction (less than 4.5 Å) – specified in each graph. The interacting residues were selected from Supplementary Table 1 as having the most frequent encounters. Mean Roll and Twist values are shown for each group. Asterisks mark the interacting ahyC residues and arrows point at the site of azide interaction.

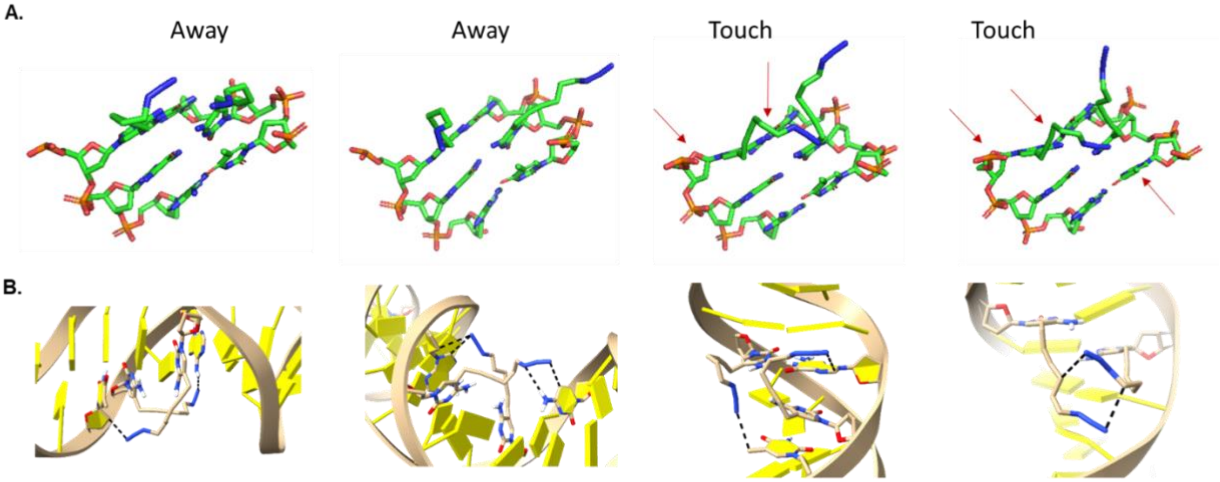

Figure S16. Representative examples of conformation fluctuations and intramolecular interactions in 4ahyC DNA (**A**) Stick-models of the 8-10th (CGA) base pairs depicting the ahyC groups exposed to solvent (Away) or touching a neighbouring base (Touch). The most prominent changes are marked with arrows. (**B**) Cartoon models of 4ahyC DNA depicting multiple simultaneous interactions of the ahyC groups.

Supplementary references

1. Strothkamp, K. G. and R. E. Strothkamp (1994). "Fluorescence Measurements of Ethidium Binding to DNA." Journal of Chemical Education 71(1): 77.

2. Smith,A.K., Wilkerson,J.W. and Knotts,T.A. (2020) Parameterization of Unnatural Amino Acids with Azido and Alkynyl R-Groups for Use in Molecular Simulations. *J. Phys. Chem. A*, **124**, 6246–6253.

3. Vanommeslaeghe, K., Hatcher, E., Acharya, C., Kundu, S., Zhong, S., Shim, J., Darian, E., Guvench, O., Lopes, P., Vorobyov, I. and Mackerell, A.D., Jr. (2010), CHARMM general force field: A force field for drug-like molecules compatible with the CHARMM all-atom additive biological force fields. J. Comput. Chem., 31: 671-690.

4. Jorgensen,W.L., Chandrasekhar,J., Madura,J.D., Impey,R.W. and Klein,M.L. (1983) Comparison of simple potential functions for simulating liquid water. *J. Chem. Phys.*, **79**, 926–935.

5. Rodrigo Galindo-Murillo, Thomas E Cheatham, III, Ethidium bromide interactions with DNA: an exploration of a classic DNA–ligand complex with unbiased molecular dynamics simulations, Nucleic Acids Research, Volume 49, Issue 7, 19 April 2021, Pages 3735–3747

6. Tribello,G.A., Bonomi,M., Branduardi,D., Camilloni,C. and Bussi,G. (2014) PLUMED 2: New feathers for an old bird. *Comput. Phys. Commun.*, **185**, 604–613.

7. Kumar,R. and Grubmüller,H. (2015) do_x3dna: a tool to analyze structural fluctuations of dsDNA or dsRNA from molecular dynamics simulations. *Bioinformatics*, **31**, 2583–2585.

8. Lu,X. and Olson,W.K. (2003) 3DNA: a software package for the analysis, rebuilding and visualization of

three-dimensional nucleic acid structures. *Nucleic Acids Res.*, **31**, 5108–5121.

9. Liutkevičiūtė, Z., Lukinavičius, G., Masevičius, V., Daujotytė D. and Klimašauskas S. (2009) Cytosine-5-methyltransferases add aldehydes to DNA. Nat Chem Biol 5, 400–402.
